# Parameter estimation in blood flow models from highly undersampled k-space magnetic resonance imaging data

**DOI:** 10.1007/s10237-026-02097-z

**Published:** 2026-06-27

**Authors:** Miriam Löcke, Pim van Ooij, Cristóbal Bertoglio

**Affiliations:** 1 Bernoulli Institute, University of Groningen, Groningen, The Netherlands; 2 Amsterdam University Medical Center, Amsterdam, The Netherlands

**Keywords:** 4D flow MRI, Inverse problems, Kalman filter, Blood flows

## Abstract

4D Flow Magnetic Resonance Imaging (MRI) is the state-of-the-art technique for measuring blood flow and provides valuable data for inverse problems in the cardiovascular system. However, acquiring 4D Flow MRI data requires long scan times, placing a burden on healthcare resources and causing discomfort for patients. To mitigate this, only part of the k-space is typically acquired, requiring additional assumptions for image reconstruction, introducing inaccuracies that can degrade the results of inverse problems. Moreover, a wide range of sampling patterns is available, and it is often unclear which one is most suitable. Here, we present a parameter estimation framework that directly uses highly undersampled k-space measurements. We solve the resulting problem numerically using a Reduced-Order Unscented Kalman Filter. We show that this approach yields more accurate estimates of boundary-condition parameters in a synthetic aortic blood flow model than approaches based on compressed-sensing reconstructions of the flow images. We also compare different sampling patterns and show how estimation accuracy depends on the sampling strategy. The results demonstrate substantially higher accuracy than inverse problems based on velocity fields reconstructed via compressed sensing. Finally, we validate these findings using real MRI data from a mechanical phantom.

## Introduction

In cardiovascular modeling, and blood flow modeling in particular, personalizing spatially distributed (i.e. 3D) models is a key step toward predictive, patient-specific simulations (Nolte and Bertoglio 2022). This requires estimating relevant model parameters from clinical data for use in diagnosis and in predictive simulations.

A common technique for measuring blood flow is phase-contrast Magnetic Resonance Imaging (MRI), or PC-MRI (Markl et al. 2003), because it is non-invasive and does not use ionizing radiation. However, acquiring 4D flow MRI data requires long scan times, which place a burden on clinical resources and on patients.

MRI applies a sequence of tissue excitations using radio-frequency (RF) pulses and modulations of the external magnetic field by gradient coils, and acquires a spatially encoded MR signal emitted by hydrogen atoms. The resulting measured complex signal corresponds to the Fourier transform of the magnetization, and its phase contains information about velocity. As such, MRI scans are acquired in k-space. One feature of MRI is that it is possible to select which k-space frequencies to measure. Therefore, to reduce the long acquisition times, often only a small part of k-space is acquired (Gottwald et al. 2020).

This partial acquisition introduces artifacts in the reconstructed image and reduces image quality. As a result, various compressed sensing (CS) techniques have been developed to reconstruct velocities from highly undersampled data while minimizing artifacts. CS has enabled greater reductions in sampling, in the sense that the accuracy of the reconstructed velocity images is higher than with previously developed sampling and reconstruction strategies (Peper et al. 2020; Lustig et al. 2007; Ye 2019). Nevertheless, as we will show later in this article, using CS-reconstructed data for parameter estimation compromises the accuracy of the estimated flow and parameters considerably.

In fact, 4D flow measurements typically involve a total number of measurements of at least $128 \times 128 \times 128 \times 20 \times 3 / R= \mathcal {O}(10^8)/R$, with R≈2-5 being the acceleration factor of the MRI acquisition in k-space. However, computational models are defined by only a few physical parameters. Therefore, we hypothesize that it is possible to reduce the number of required k-space measurements without compromising the accuracy of the parameter estimation.

Specifically, we introduce a new technique for estimating blood flow parameters directly from undersampled k-space MRI data. By bypassing the reconstruction/compressed sensing step, this approach both avoids reconstruction artifacts and improves computational efficiency. Preliminary results in this direction were reported in Löcke et al. (2025), but they were not explored in depth. In this paper, we provide additional methodological details, present further results for different $V_{enc}$ values and for individual parameters, and examine the robustness of the method when the magnitude and background phase are approximated. We also present results on real MRI phantom data.

The remainder of the paper is structured as follows. In Sect. 2, we introduce the theory of MRI measurements and reconstruction, as well as the formulation of the inverse problem of parameter estimation in blood flow models. In Sect. 3, we describe the setup of the numerical examples used to study the performance of the proposed method using synthetic and real data, and in Sect. 4 we present the results. Finally, in Sect. 5, we discuss the implications of our findings and potential future directions.

## Theory

### MRI measurement and reconstruction in a nutshell

#### Velocity encoding

Let us denote by $u(\boldsymbol{x}, t)$ the component of the velocity $\boldsymbol{u}(\boldsymbol{x}, t)$ in the direction $\boldsymbol{d}$ (fixed in space and in time), and $\boldsymbol{x}\in \mathbb {R}^3$, $t\in \mathbb {R}$ represent the spatial and temporal location within the image space, respectively. The MR images usually analyzed in the clinical context are actually complex valued, namely the so-called *transverse magnetization*^1^

1 $$\begin{aligned} m(\boldsymbol{x}, t) =M(\boldsymbol{x}, t) \exp {(i\phi (\boldsymbol{x}, t))} \in \mathbb {C}, \end{aligned}$$

where the magnitude M>0 usually displays the anatomy, and the phase $\phi (\boldsymbol{x}, t) \in (-\pi , \pi ]$ in blood flow imagining takes the form

2 $$\begin{aligned} \phi (\boldsymbol{x}, t) = \frac{\pi }{venc}u(\boldsymbol{x}, t) + \phi _{back}(\boldsymbol{x}, t) \end{aligned}$$

where $\phi _{back}$ is the background phase, and $venc\in \mathbb {R}$ is the selected velocity encoding (which is a function inversely proportional to the strength and duration of the velocity encoding magnetic gradients).

Since both *u* and $\phi _{back}$ are unknown at every voxel, at least two measurements are required. In the simplest case (which is the most commonly used in clinical practice), one measurement with no velocity encoding gradient is acquired to obtain $\phi _{back}$, and a second one with venc≠0, leading to two complex magnetic measurements, so that the velocity can be simply obtained by subtracting the phases, namely,

3 $$\begin{aligned} \hat{u} = \frac{\phi - \phi _{back}}{\pi }venc. \end{aligned}$$

Therefore, this technique is called *Phase-Contrast MRI (PC-MRI)*.

In practice, the image $m(\boldsymbol{x}, t)$ is discrete in both space and time, being divided into voxels and time instants. A *voxel* refers to a spatial unit across which the signal is assumed to be constant to provide a single value. The number of voxels is determined by the spatial resolution. The time instants are the times at which a measurement is made, which are determined by the temporal resolution. Therefore $m(\boldsymbol{x}, t)$ can be represented as a matrix in $\mathbb {C}^{N_x\times N_y \times N_z \times N_T}$, with $N_x, N_y, N_z$ being the number of voxels in each spatial direction and $N_T$ the number of time instants. We will refer to the total number of voxels as $N = N_x \cdot N_y \cdot N_z$.

#### The raw signal and image reconstruction

The raw signal measured by the MRI scanner corresponds to the spatial Fourier transform of the magnetization, which can, at each measured “time instant", be formulated as

4 $$\begin{aligned} \boldsymbol{Y}(\boldsymbol{k}) = \mathcal {F}\left[ \boldsymbol{M}\odot \exp \left( i\left( \frac{\pi }{venc}\boldsymbol{u}_{meas} + \phi _{back}\right) \right) \right] (\boldsymbol{k}) + \epsilon (\boldsymbol{k}) \end{aligned}$$

where ⊙ denotes the Hadamard product. We now consider discrete quantities $\boldsymbol{M} \in \mathbb {R}^N$, $\boldsymbol{u}_{meas}\in \mathbb {R}^N$ and $\mathcal {F}: \mathbb {R}^N \rightarrow \mathbb {R}^N$ is the three-dimensional discrete Fourier transform defined as

5 $$\begin{aligned} \mathcal {F}[\boldsymbol{X}](\boldsymbol{k})= & \sum _{n_x=0}^{N_x-1}\sum _{n_y=0}^{N_y-1}\sum _{n_z = 0}^{N_z - 1} \boldsymbol{X}_{n_xn_yn_z} \nonumber \\ & \exp \left( -i2\pi \left( \frac{k_1n_x}{N_x} + \frac{k_2n_y}{N_y} + \frac{k_3n_z}{N_z}\right) \right) \end{aligned}$$

and $\boldsymbol{\epsilon } (\boldsymbol{k})\in \mathbb {C}^N$ is a complex Gaussian noise with a mean of zero(Irarrazaval et al. 2019). Hence the measurements are $\boldsymbol{Y}^n \in \mathbb {C}^N$ for $n = 1, \cdots , N_T$. Due to commonly using the notation (k), the frequency space associated to the MR image acquisition is typically called the k-space.

In practice, for a given temporal resolution, only a limited number of k-space lines can be acquired. As a result, the MRI scanner acquires different k-space lines during each cardiac cycle. Consequently, a “time instant” *n* actually contains frequencies measured over different cardiac cycles, under the assumption that there are no significant differences between consecutive cycles (or, if there are differences, for instance in the heart rate, the corresponding data are rejected). In principle, one could also choose different k-space trajectories at different instants of the cardiac cycle, although this is rarely done.

The noise level depends on the scan parameters. For instance, lower spatial resolution (i.e., larger voxel size) increases the signal-to-noise ratio since the signal increases while the device noise remains constant. Similarly, a lower *venc* increases the sensitivity of the magnetization phase to velocity variations, increasing the *velocity-to-noise ratio* (Carrillo et al. 2018). However, since the phase is constrained to the range $[-\pi , \pi )$, the velocity is limited to [-venc,venc). If the maximum velocity exceeds the chosen *venc*, phase aliasing artifacts occur. Thus, selecting an appropriate *venc* requires balancing noise levels against aliasing artifact presence.

*Undersampling and reconstruction.* If the k-space is fully sampled, i.e. the values of all k-space locations are known, the velocity can be reconstructed by applying the inverse Fourier transform to $\boldsymbol{Y}^n$ and equation (3). However, fully sampling the k-space is not practicable in cardiovascular MRI scans since 3D scans may take of the order of hours, depending on the spatiotemporal resolution chosen.

Therefore, to reduce the length of the scan, the k-space is generally undersampled. There are many k-space sampling patterns used in MRI applications, usually in combination with a reconstruction algorithm specific to the sampling pattern.

Some common 2D patterns include regular Cartesian sampling, radial sampling, and pseudo-spiral sampling (Feng 2022). These can be extended to 3D by stacking 2D designs, leading to stack-of-stars and stack-of-spirals designs (Feng 2022). The patterns can differ in whether lines are arranged horizontally and vertically or in a shear pattern, in the number of radial lines or spiral arms, in the number of points per line, and in whether the patterns are stacked with equidistant or variable density in the z-direction. Additionally, in stack-of-stars and stack-of-spirals designs, the pattern can be rotated by a certain angle at each level in the stack.

Another option is to use pseudo-random sampling methods, usually with a higher density in the center, to ensure that the artifacts are incoherent and resemble noise (Peper et al. 2020). Here as well, there are many choices for the probability distribution to use.

Undersampled data cannot be reconstructed with an inverse Fourier transform, as it violates the Nyquist limit. *Compressed sensing* (CS) techniques are commonly used to reconstruct undersampled data in a way that limits the presence of artifacts. Generally this can be formulated as solving the optimization problem

6 $$\begin{aligned} \boldsymbol{\hat{m} = }\text {argmin}_{\boldsymbol{m}}\{||\mathcal {F}_U(\boldsymbol{m}) - \boldsymbol{Y}||^2_2 + \lambda ||\Phi \boldsymbol{m}||_1\} \end{aligned}$$

where $\mathcal {F}_U$ is the Fourier transform combined with the undersampling, $\boldsymbol{Y}$ is the measured k-space data, $\boldsymbol{\hat{m}}$ is the reconstructed image data, Φ is a sparsifying transform, and λ is the regularization parameter.

The sparsifying transform maps the image to a domain where the signal has a sparse representation. In MRI, common choices include wavelet transforms and temporal total variation, depending on the data’s spatio-temporal structure and the features to be preserved. In compressed sensing, pseudo-random sampling masks are preferred over regular undersampling because they distribute aliasing energy as incoherent, noise-like interference rather than coherent, structured ghosts. Because this incoherent artifact is diffuse, the sparsifying transform can effectively distinguish the true, sparse signal coefficients from the background interference during reconstruction. The regularization parameter λ balances this denoising process and is typically chosen empirically to optimize image quality.

### The k-space parameter estimation problem

#### Parameters estimation in PDEs

To consider the underlying physics of the the application, we assume that they can be modeled as the solution $\boldsymbol{u}: \mathbb {R}^3\times [0, T] \rightarrow \mathbb {R}^3$ of a partial differential equation

7 $$\begin{aligned} F\left( \boldsymbol{u}, \frac{\partial \boldsymbol{u}}{\partial x_1}, \frac{\partial \boldsymbol{u}}{\partial x_2}, \frac{\partial \boldsymbol{u}}{\partial x_3}, \frac{\partial \boldsymbol{u}}{\partial t}, \boldsymbol{\theta }\right) = 0 \end{aligned}$$

in a domain $\Omega \subset \mathbb {R}^3$ with an initial condition $\boldsymbol{u}(\boldsymbol{x}, 0) = \boldsymbol{u}^0$ and a set of boundary conditions which is dependent on a set of parameters $\boldsymbol{\theta }$, which can describe boundary conditions or material parameters. Then we can define the forward operator $\mathcal {A}(\boldsymbol{\theta })$ which describes the solution of this PDE for the vector of model parameters $\boldsymbol{\theta }\in \mathbb {R}^p$. As such, the forward problem generates data according to a physical model with given model parameters.

The goal of the inverse problem overlying this PDE is to estimate a (sub)set of the parameters $\boldsymbol{\theta }$ given measurements of $\boldsymbol{u}(\boldsymbol{x}, t)$.

#### Measurements

For formulating the inverse problem to estimate the parameters $\boldsymbol{\theta }$, we have three different choices for the measurements:

1. the velocity data $\boldsymbol{V}^n \in \mathbb {R}^N$ for $n=1, \cdots , N_T$. $\boldsymbol{V}^n$ is calculated from the angle of the magnetization, meaning that in the case of undersampling the magnetization has to be reconstructed first using compressed sensing as well. Additionally, the noise on this measurement cannot be approximated well by a Gaussian distribution (Irarrazaval et al. 2019).
2. the k-space data $\boldsymbol{Y}^n \in \mathbb {C}^N$ for $n = 1, \cdots , N_T$, which might be undersampled. The noise on this data is Gaussian, independently distributed, and zero-mean.
3. the magnetization data $\boldsymbol{M}_{meas}^n \in \mathbb {C}^N$ for $n=1, \cdots , N_T$, which relates to the frequency data as $\boldsymbol{M}^n_{meas} = \mathcal {F}^{-1}(\boldsymbol{Y}^n) = \boldsymbol{M}_{true} + \mathcal {F}^{-1}(\boldsymbol{\epsilon })$. This is also complex-valued data with a Gaussian noise with zero mean, as the inverse Fourier transform of a Gaussian is Gaussian as well. However, if the k-space is undersampled, this measurement has to be reconstructed with CS. In this case, the noise on the velocity measurements cannot be assumed to be Gaussian and independently distributed, as there are correlations between the noise in different pixels (Partin et al. 2022).

For all three types of measurements, each velocity direction is acquired separately, therefore it is possible that only one or two velocity components are available rather than all three, or that they are acquired in a direction that does not match a vector component in the canonical basis.

#### Objective function

In a Bayesian framework, the inverse problem for the estimation of parameters from velocity measurements can be solved by minimizing the functional

8 $$\begin{aligned} \boldsymbol{\hat{\theta }} = \text {argmin}_{\boldsymbol{\theta } \in \mathbb {R}^p} \frac{1}{2\sigma _v^2}\sum _{n=1}^{N_T}\sum _{s=1}^N \left( \left[ \boldsymbol{V}^n - \mathcal {H}(\boldsymbol{u}_{\boldsymbol{\theta }}^n)\right] _s\right) ^2 + \frac{1}{2} ||\boldsymbol{\theta } - \boldsymbol{\theta }^0||_{(\boldsymbol{P}^0)^{-1}}^2 \end{aligned}$$

where $\boldsymbol{V}^k \in \mathbb {R}^N$ are the measurements of the velocity provided by the reconstruction of the PC-MRI acquisition and $\mathcal {H}: [H_1(\Omega )]^3 \rightarrow \mathbb {R}^N$ is the observation operator, which is applied to the result $\boldsymbol{u}_{\boldsymbol{\theta }}^n$ of the forward model for a set of model parameters $\boldsymbol{\theta }\in \mathbb {R}^p$ at the time corresponding to the measurement *n*. In this standard inverse problem for velocities, this observation operator usually corresponds to the interpolation from the model’s (usually finer) mesh to an array of measured velocities at each image voxel. In fact, $[.]_s$ denotes the *s*-th vector element (or voxel in the case of image data), therefore summing over all the vector elements. $\boldsymbol{\theta }^0$ is the initial guess for the parameters with its covariance matrix $\boldsymbol{P}^0$, both of which are given by the user but are assumed to be known for the numerical inverse solver. $\sigma _v$ denotes the standard deviation of the noise on the velocity measurements, which we also assume to be (approximately) known.

This inverse problem has been commonly applied to the estimation of boundary conditions in hemodynamics. For a thorough review, see Nolte and Bertoglio (2022).

In Garay et al. (2022), an alternative formulation of this objective function was proposed to account for aliasing artifacts present in velocity MRI data. The functional in this case is

9 $$\begin{aligned} \boldsymbol{\hat{\theta }}= & \text {argmin}_{\boldsymbol{\theta } \in \mathbb {R}^p} \frac{1}{2 \sigma _M^2} \sum _{n=1}^{N_T}\sum _{s=1}^N \Big (\Big ( [\Re (\boldsymbol{M}_{meas}^n) \nonumber \\ & - |\boldsymbol{M}_{meas}^n|\cos (\phi _{back} + \mathcal {H}(\boldsymbol{u}_{\boldsymbol{\theta }}^n)\frac{\pi }{venc})]_s \Big )^2 \nonumber \\ & + \Big ([ \Im (\boldsymbol{M}_{meas}^n) - |\boldsymbol{M}_{meas}^n|\sin (\phi _{back} + \mathcal {H}(\boldsymbol{u}^n_{\boldsymbol{\theta }})\frac{\pi }{venc} ]_s) \Big )^2\Big ) \nonumber \\ & + \frac{1}{2} ||\boldsymbol{\theta } - \boldsymbol{\theta }^0||_{(\boldsymbol{P}^0)^{-1}}^2 \nonumber \\= & \text {argmin}_{\boldsymbol{\theta } \in \mathbb {R}^p} \sum _{n=1}^{N_T}\sum _{s=1}^N \frac{|\boldsymbol{M}_{meas}^n|_s^2}{\sigma _M^2} \Big ( 1 - \cos (\frac{\pi }{venc}([\boldsymbol{V}^n - \mathcal {H}(\boldsymbol{u}^n_{\boldsymbol{\theta }})))]_s \Big )\nonumber \\ & + \frac{1}{2} ||\boldsymbol{\theta } - \boldsymbol{\theta }^0||_{(\boldsymbol{P}^0)^{-1}}^2 \end{aligned}$$

where $\boldsymbol{M}^n_{meas}$ are measurements of the complex magnetization and $\sigma _M$ is the standard deviation of the noise for each of the (complex magnetization) real/imaginary measurements. This formulation assumes that the magnitude $|\boldsymbol{M}^n_{meas}|$ of the magnetization is known in order to formulate the problem in terms of the measured and observed velocities.

However, the aforementioned cost functions do not account for artifacts arising from frequency undersampling of the data. Therefore, as we will observe in the numerical examples, the error in the data, and consequently in the parameters estimated through the inverse problem, grows drastically as the degree of undersampling increases. Here, we propose to address this issue by formulating the parameter estimation problem, as is done in CS, using a data-fidelity term defined in k-space, leading to the following minimization problem:

10 $$\begin{aligned} \boldsymbol{\hat{\theta }}= & \text {argmin}_{\boldsymbol{\theta } \in \mathbb {R}^p} \frac{1}{2\sigma _y^2}\sum _{n=1}^{N_T}\sum _{s=1}^N ([\Re (\boldsymbol{Y}^n - \mathcal {H}_{\mathcal {F}}(\boldsymbol{u}_{\boldsymbol{\theta }}^n))]_s)^2 + ([\Im (\boldsymbol{Y}^n - \mathcal {H}_{\mathcal {F}}(\boldsymbol{u}_{\boldsymbol{\theta }}^n))]_s)^2 \nonumber \\ & + \frac{1}{2} ||\boldsymbol{\theta } - \boldsymbol{\theta }^0||_{(\boldsymbol{P}^0)^{-1}}^2 \end{aligned}$$

with the observation operator $\mathcal {H}_{\mathcal {F}}$ being defined as

11 $$\begin{aligned} {{\mathcal H}_{\mathcal {F}}}(u) = {\mathcal F}\left( {\boldsymbol{M}}\odot e^{i\frac{\pi }{venc}{\boldsymbol{u}} + \phi _{back}}\right) \odot {\boldsymbol{S}} \end{aligned}$$

where $\boldsymbol{S} \in \mathbb {R}^N$ is the sampling mask, i.e. with the entries corresponding to the sampled voxels set to 1, and the others to 0. The $\boldsymbol{Y}^n$ are measurements in k-space and $\sigma _y$ is the standard deviation of the noise on the frequency-space measurements, which is assumed to be Gaussian and zero-mean. The use of this observation operator requires that the magnitude of the magnetization as well as the background phase, or an approximation thereof, is known.

#### Parameter estimation with Kalman filtering

Various approaches can be used to solve the optimization problems described above. Adjoint-based variational data assimilation methods fit the full set of measurements to the corresponding observations at matching time steps. However, this entails significant storage requirements, as the entire trajectory must be stored. In contrast, sequential data assimilation incorporates the information from each new measurement when it becomes available during a single forward pass of the model. This leads to a sequential improvement of the estimate over the course of the inverse problem as more measurements are incorporated.

We have chosen to use the Reduced Order Unscented Kalman Filter (ROUKF)(Moireau and Chapelle 2011) in order to solve the optimization problem (10), as it is computationally tractable and has been successfully used in blood-flow and other time-resolved problems due to its recursive nature. In this context, ROUKF has previously been employed successfully for parameter estimation from already reconstructed MR images, but not, to our knowledge, from raw k-space data (Arthurs et al. 2020; Imperiale et al. 2021).

ROUKF is a sequential parameter estimator which corrects the posterior distribution of the state and parameters in each time step with the available measurement. To do so, ROUKF generates a fixed number of particles sampled from the prior distribution and propagates each of them through the forward problem. The propagated state of each particle is then used to compute the correction of the mean and covariance of the parameters whenever measurements are available.

As a result, the filter relies on the measurement error or *innovation*, which is proportional to the derivative of the data fidelity term of the objective function with respect to *u*. For the objective function (8), this leads to the innovation

12 $$\begin{aligned} \boldsymbol{\Gamma }^n = \boldsymbol{V}^n - \mathcal {H}(\boldsymbol{u}^n) \end{aligned}$$

Similarly, for the objective function in (9), the innovation results from the derivative of the cost function with respect to the state, namely

13 $$\begin{aligned} \boldsymbol{\Gamma }^n = \frac{1}{\sqrt{2}}|\boldsymbol{M}(t^n)| \sin \left( \frac{\pi }{venc} \cdot \left( \boldsymbol{V}^n - \mathcal {H}(\boldsymbol{u}^n)\right) \right) \end{aligned}$$

where the factor $\frac{\sqrt{2}venc}{\pi }|\boldsymbol{M}(t^n)|^{-1}$ is added to ensure the equivalence of this innovation with Equation (12) in the case of high *venc*. For more details, see Garay et al. (2022).

As done for the first cost function, assuming a Gaussian distribution in the noise of the complex magnetization measurements, the innovation for the k-space based objective function (10) is defined by:

14 $$\begin{aligned} \boldsymbol{\Gamma }_n = \begin{bmatrix} \Re (\boldsymbol{Y}^n) - \Re (\mathcal {H}_{\mathcal {F}}(\boldsymbol{u}^n))\\ \Im (\boldsymbol{Y}^n) - \Im (\mathcal {H}_{\mathcal {F}}(\boldsymbol{u}^n)) \end{bmatrix}. \end{aligned}$$

Moreover, we note that we adapt the Kalman filter as described in Moireau and Chapelle (2011). Instead of simplex sigma points (which involve p+1 solutions of the forward problem), we use canonical sigma points, which involve 2*p* solutions of the forward problem but have shown better performance in our simulations than their simplex counterparts. In particular, the discrepancy between the results obtained with the two types of sigma points increases with the number of parameters to be estimated. In addition, the results with simplex points depend on the ordering of the parameters during the estimation process, whereas this is not the case with canonical points.

Additionally, to ensure positivity of the parameters, we are reparameterizing such that $\boldsymbol{\theta } = \boldsymbol{\theta }^0 2^\nu$ with $\boldsymbol{\theta }^0$ the initial guess for the parameters and the filter being applied to ν, as is also done in Garay et al. (2022). The initial value of ν is set to $\boldsymbol{0}$.

## Methods

### Synthetic data

#### Forward problem setup

**Fig. 1:**
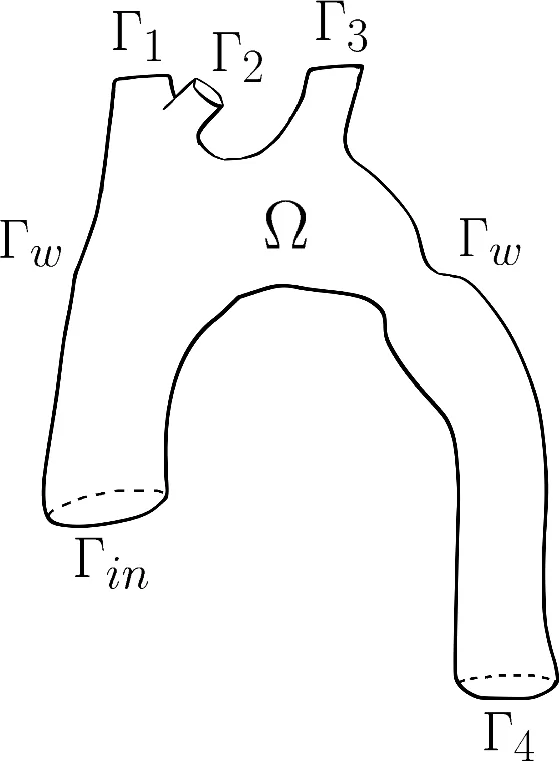
3D aortic model geometry used for the forward problem. The inlet is located in the ascending aorta, and the outlets are located in the descending aorta and the three branches of the aortic arch

We use the same model as in Garay et al. (2022). We consider the lumen geometry of the ascending and descending aorta, including the outlets of the brachycephalic artery, left common carotid artery, and left subclavian artery, as depicted in Fig. 1. This geometry serves as the computational domain for the forward model. It was discretized using unstructured trapezoidal elements, with a total of 20,916 points.

The boundary of the geometry consists of six different boundaries: $\Gamma _{in}$ being the inlet boundary in the ascending aorta, $\Gamma _{w}$ the arterial wall, and the remaining boundaries $\Gamma _l$ for l=1,⋯,4 representing the outlets.

We model the blood flow in this domain with the incompressible Navier–Stokes equations for the velocity $\boldsymbol{u}(\boldsymbol{x}, t)\in \mathbb {R}^3$ and the pressure $p(\boldsymbol{x},t)\in \mathbb {R}$:

15a $$\begin{aligned} \rho \frac{\partial \boldsymbol{u}}{\partial t} + \rho (\boldsymbol{u} \cdot \nabla )\boldsymbol{u} - \mu \Delta \boldsymbol{u} + \nabla p = 0 \text { in } \Omega \end{aligned}$$

15b $$\begin{aligned} \nabla \cdot \boldsymbol{u} = 0 \text { in } \Omega \end{aligned}$$

15c $$\begin{aligned} \boldsymbol{u} = \boldsymbol{u}_{in} \text { on } \Gamma _{in}\end{aligned}$$

15d $$\begin{aligned} \boldsymbol{u} = \boldsymbol{0} \text { on } \Gamma _w\end{aligned}$$

15e $$\begin{aligned} \mu \frac{\partial \boldsymbol{u}}{\partial \boldsymbol{n}} - p\boldsymbol{n} = -P_l(t)\boldsymbol{n} \text { on } \Gamma _l \end{aligned}$$

with $\rho , \mu$ the density and dynamic viscosity of the fluid and $P_l(t)$ being given by a Windkessel boundary condition defined by:

16a $$\begin{aligned} P_l = R_{p,l}Q_l + \pi _l\end{aligned}$$

16b $$\begin{aligned} Q_l = \int _{\Gamma _l} \boldsymbol{u} \cdot \boldsymbol{n} dS\end{aligned}$$

16c $$\begin{aligned} C_{d, l} \frac{d\pi _l}{dt} + \frac{\pi _l}{R_{d, l}} = Q_l. \end{aligned}$$

This boundary condition models the effects of the remaining vascular system on the outlet via the proximal and distal resistances $R_p$, $R_d$ of the vasculature and the distal compliance $C_d$ of the vessels.

The inflow $\boldsymbol{u}_{in}$ is defined as

17 $$\begin{aligned} \boldsymbol{u}_{in} = -U f(t) \boldsymbol{n} \end{aligned}$$

where *U* is a constant amplitude and

18 $$\begin{aligned} f(t) = {\left\{ \begin{array}{ll} \sin (\frac{\pi t}{T}) \text { if } t \le T\\ \frac{\pi }{T}(t-T)\exp ^{-\kappa (t-T)} \text { if } T_c> t > T \end{array}\right. } \end{aligned}$$

with $$T_c = 0.8$$ and T=0.36.

The physical parameters are set as described in Table 1.

**Table 1:** Physical parameters and numerical values of the three-element Windkessel parameters for every outlet used in the forward simulation

Parameter	Value
$\rho \ (gr \cdot cm^3)$	1.2
$\mu \ (P)$	0.035
$U \ (cm \cdot s^{-1})$	75
$T_c \ (s)$	0.80
T(s)	0.36
$\kappa \ (s^{-1})$	70

	$\Gamma _{1}$	$\Gamma _{2}$	$\Gamma _{3}$	$\Gamma _{4}$
$R_p \ (dyn \cdot s \cdot cm^{-5})$	480	520	520	200
$R_d \ (dyn \cdot s \cdot cm^{-5})$	7200	11520	11520	4800
$C \ (dyn^{-1} \cdot cm^5 )$	$4\cdot 10^{-4}$	$3\cdot 10^{-4}$	$3\cdot 10^{-4}$	$4\cdot 10^{-4}$

The forward problem is solved using an in-house finite elements solver, with a semi-implicit 3D-0D coupling scheme as in Garay et al. (2022) and using *P*1 elements for both the velocity and the pressure (Guermond 1996). The full algorithm is detailed in the appendix.

#### Synthetic measurements

The forward solution is generated with a time step of $dt = 1\,\textrm{ms}$ and then undersampled in time to $dt_{meas} = 15\,\textrm{ms}$, resulting in a total of 56 measurements. From this solution, we simulate a PC-MRI acquisition by subsampling onto a rectangular measurement mesh with a resolution of $[2\,\textrm{mm}, 2\,\textrm{mm}, 2\,\textrm{mm}]$ and then applying the process described in Sect. 2.1 with a *venc* equal to twice the maximum velocity. The magnitude is modeled as

19 $$\begin{aligned} M(\boldsymbol{x}) = {\left\{ \begin{array}{ll} 1.0 \text { if } \boldsymbol{x} \text { is in the lumen of the vessel}\\ 0.5 \text { otherwise.} \end{array}\right. } \end{aligned}$$

and the background phase was set to an arbitrary constant value of $\phi _{back} = 7.5\cdot 10^{-2}rad$. Finally a complex Gaussian noise $\boldsymbol{\epsilon } \in \mathbb {C}^N$ is added with a signal-to-noise ratio (SNR) of 15. Fifty independent realizations of the noise were generated.

For comparison, we reconstructed velocity measurements from these synthetic data using the Berkeley Advanced Reconstruction Toolbox (BART) (Blumenthal et al. 2023). BART is a command-line-based software package that provides a flexible framework for compressed sensing methods, as well as tools for simulation, pre-processing, and image reconstruction, with a wide range of regularization options. In this work, we used this toolbox to perform compressed sensing reconstructions of the velocity, using total variation in time for regularization. Examples of the reconstructed velocities obtained with different masks and acceleration factors are shown in Fig. 4.

**Fig. 2:**
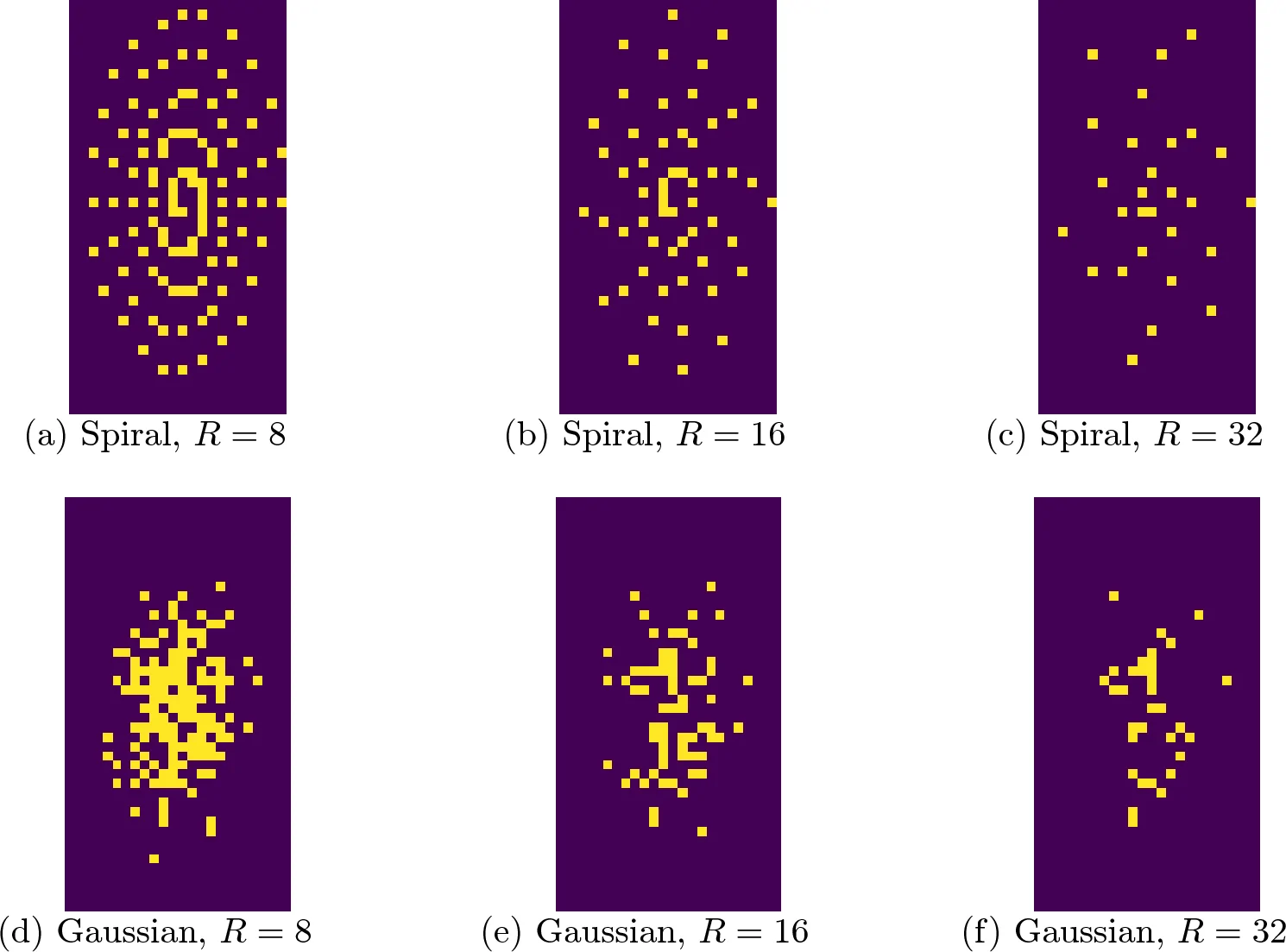
Sampling masks used for the undersampling of the k-space measurements. The spiral mask leads to more structured artifacts, whereas the Gaussian mask leads to more incoherent, noise-like artifacts

Next, the sampling mask is applied to these simulated k-space measurements. We take a 2D subsampled mask in the x-y-plane and sample fully in the *z*-direction as in Peper et al. (2020). We consider different subsampling rates $R = \frac{N_{sampled}}{N_{total}} = {8, 16, 32}$, with two different masks: the pseudo-spiral mask and the pseudo-random Gaussian mask, which is sampled according to a Gaussian probability distribution, as shown in Fig. 2. For the pseudo-spiral mask, the points are placed evenly on a cartesian grid along a spiral with six turns and a final radius reaching the edge of the mask.

Additionally, we require measurements of the magnitude itself. The value of the background phase is treated as known instead.

**Fig. 3:**
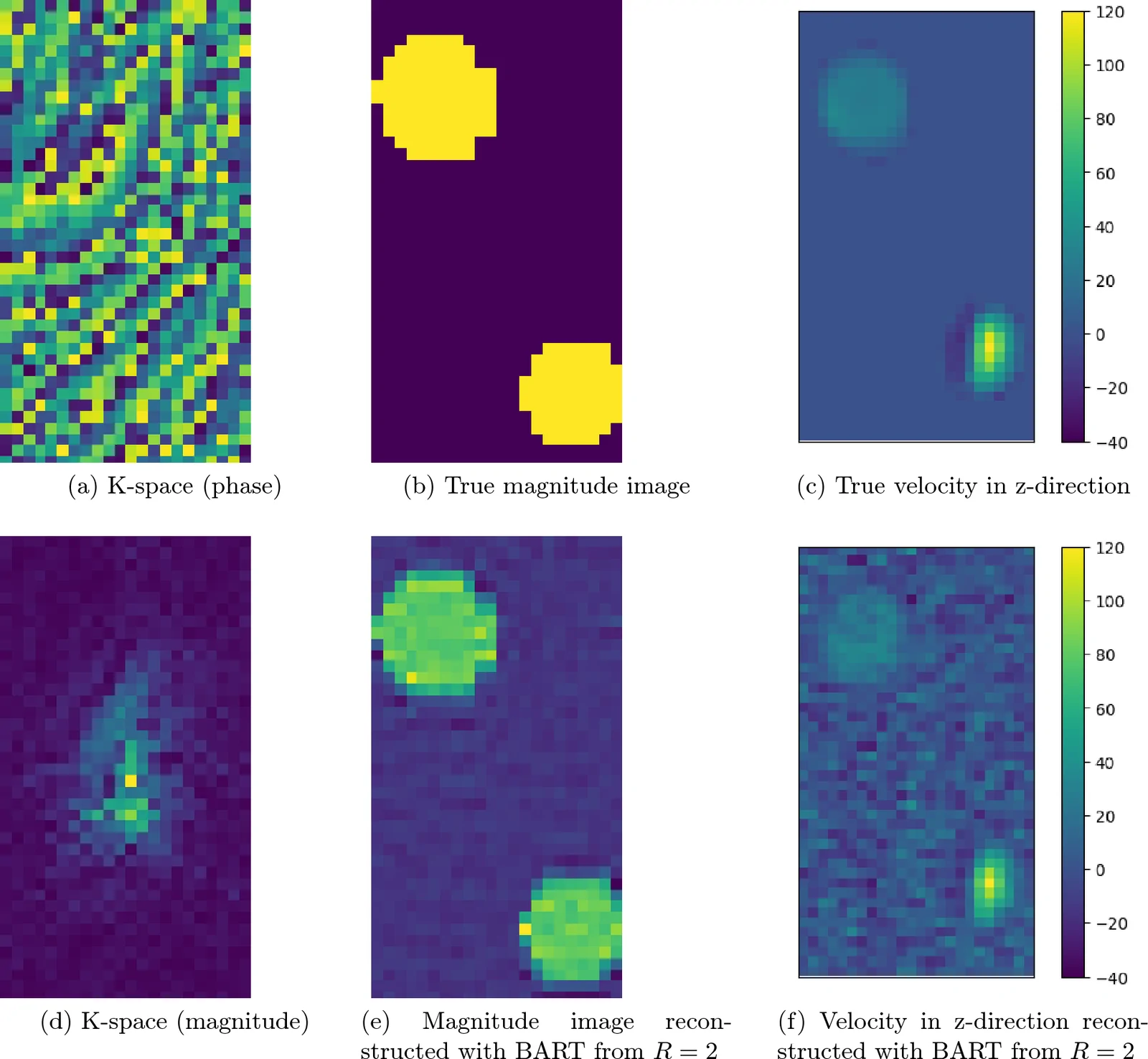
Examples of simulated measurements, taken at a slice in the *z*-direction. The first row shows the phase of the k-space measurements, the true magnitude of the magnetization, and the true velocity in the z-direction. The second row shows the magnitude of the k-space measurements, the magnitude of the magnetization reconstructed with BART from R=2, and the velocity in the z-direction reconstructed with BART from R=2. The magnitude reconstructed with BART from R=2 is used as the magnitude measurement for the inverse problem

As anatomical images are usually readily available and the magnitude generally does not depend on the encoding direction, making a cheap 2D/3D acquisition feasible, we consider a reconstructed magnitude from the measurements with R=2 and a Gaussian mask using temporal *l*1-regularization in BART with a regularization parameter λ=0.001. Examples of the phase and magnitude of the measurements are shown in Fig. 3.

**Fig. 4:**
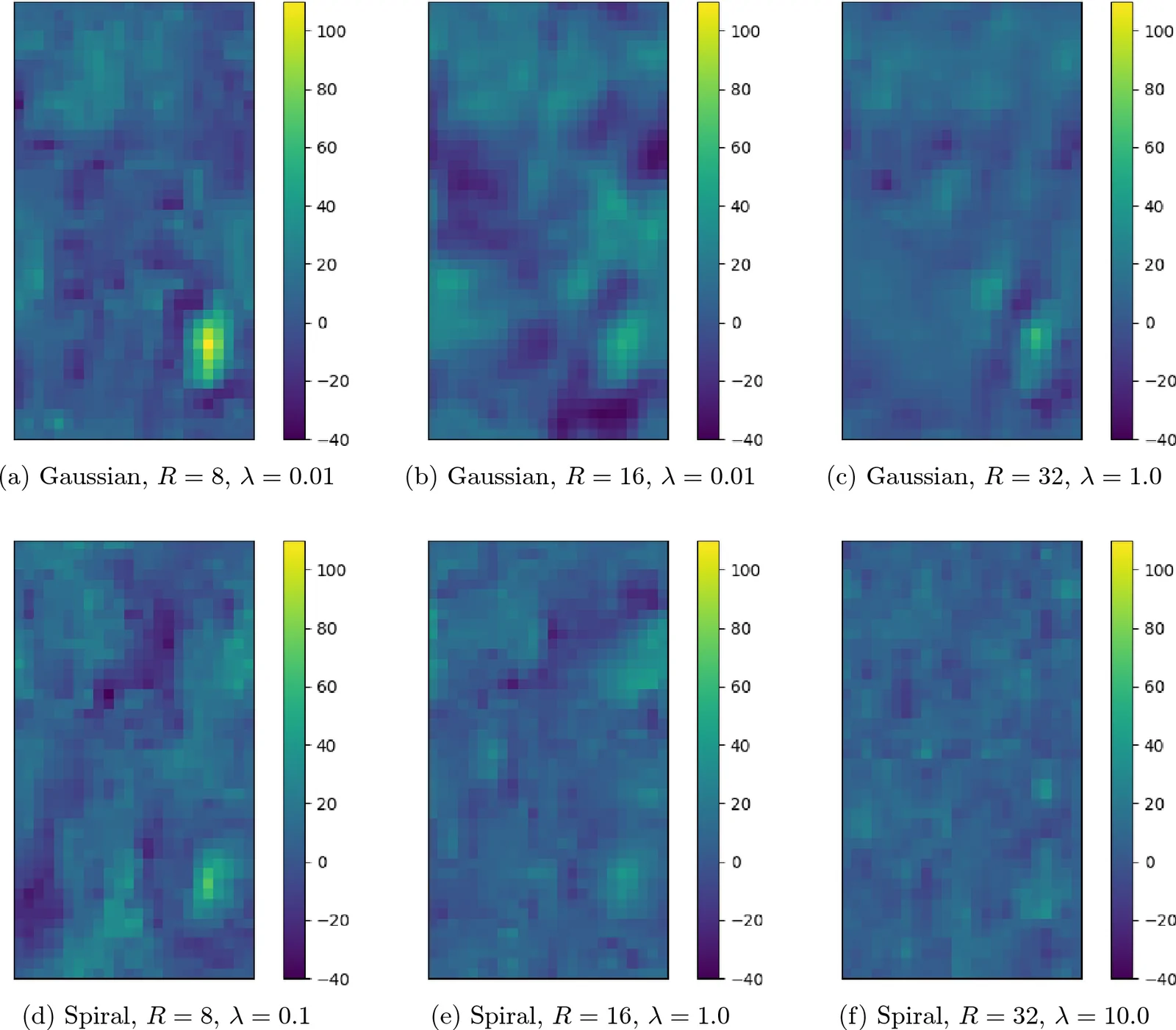
Examples of velocities reconstructed with BART using Gaussian and Spiral sampling masks and different acceleration factors. Depending on the sampling mask, different kinds of artifacts appear in the reconstructed velocity

#### Inverse problem setup

Without pressure measurements, not all the Windkessel resistances in the system can be uniquely determined at once. Therefore we have fixed the values for $\Gamma _4$ (the outlet in the descending aorta), and estimate the amplitude of the inflow *U* as well as the distal resistances $R_{d,k}$, k=1,2,3 of the remaining Windkessel boundary conditions.

We consider two different initial guesses for these parameters, as in Garay et al. (2022):

1. "low" guess: U=40, $$R_{d,1}=4000$$, $$R_{d,2}=4000$$, $$R_{d,3}=4000$$
2. "high" guess: U=150, $$R_{d,1}=20000$$, $$R_{d,2}=20000$$, $$R_{d,3}=20000$$

while the target values are U=75, $R_{d,1}=7200$, $R_{d,2}=11520$, $R_{d,3}=11520$.

The initial standard deviation for the reparameterized parameters was set to 0.5, i.e. $P^0 = 0.5\mathbb {I}$, meaning that the prior models that there is $\approx 95\%$ probability that the target value will lie within the range of half/twice the initial guess. The standard deviation of the noise was estimated from the initial time step by computing the standard deviation of

20 $$\begin{aligned} \boldsymbol{Y}^0 - \boldsymbol{M}(t^0)\odot \exp \left( i\phi _{back}(t^0))\right) \odot \boldsymbol{S} \end{aligned}$$

which is the noise of the data under the assumption that the velocity at the first time step is zero. The estimated standard deviation for each case is shown in Table 2. The estimation of the noise in this case is impacted by noise in the reconstructed magnitude.

**Table 2:** Estimated standard deviation of the noise for each mask and acceleration factor, using the magnitude reconstructed from R2 for acceleration factors greater than 1

Acceleration factor	Gaussian	Spiral
R1/actual value	15.526	
R8	15.829	13.813
R16	18.716	13.791
R32	23.043	13.844

### Phantom data

#### Flow phantom measurements

We used data from a flow experiment on a phantom of the carotid artery reported in Peper et al. (2020). The phantom is made of a distensible silicon and is suspended in water. It takes the shape of a bifurcating tube, to simulate a carotid artery, and a backflow tube. A pump generates a pulsatile flow with a rate of roughly 60 bpm, simulating a cardiac cycle lasting 1 s.

**Fig. 5:**
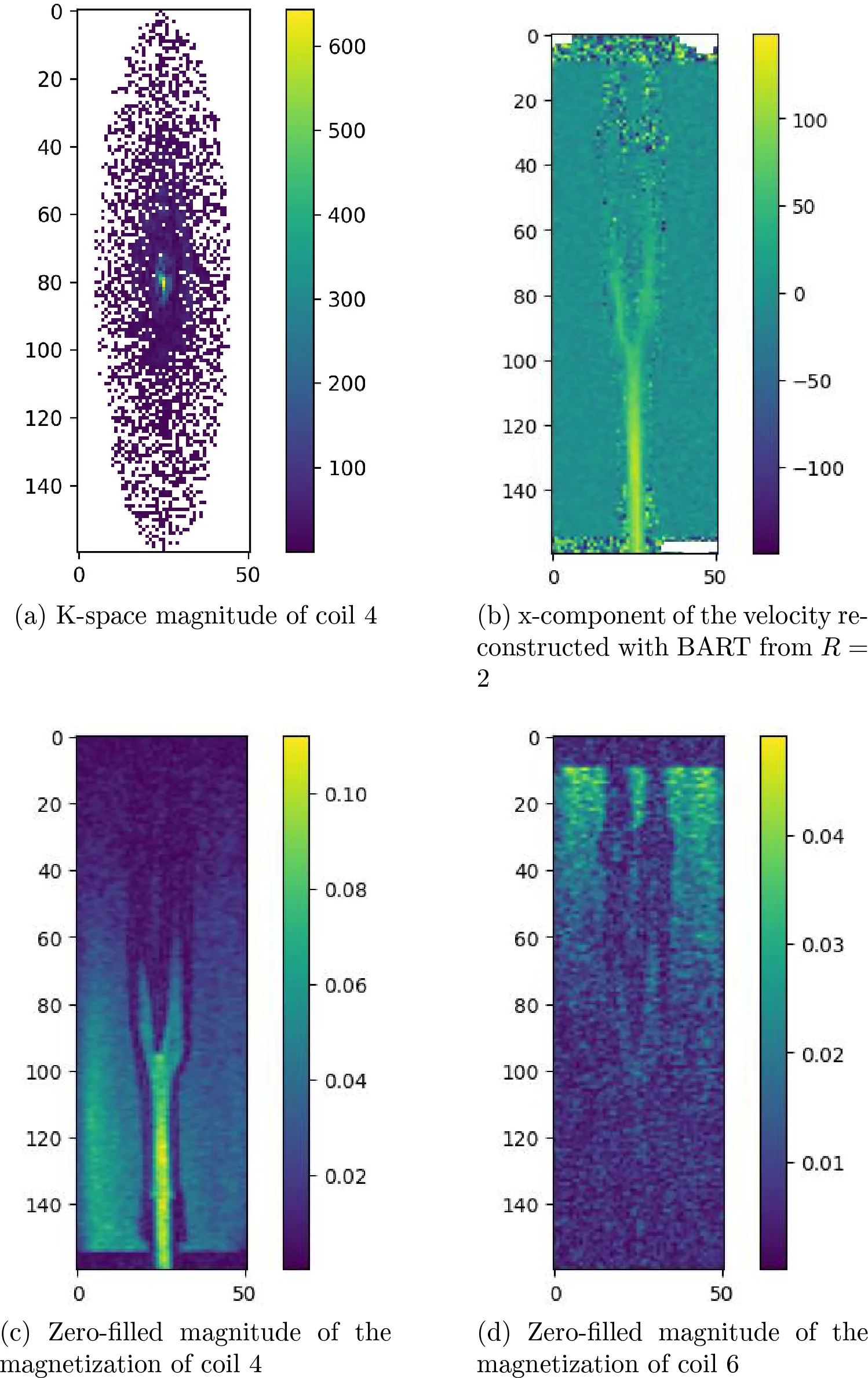
Examples of the phantom measurements. Reconstructed magnitudes of the magnetization of different coils show the different coil sensitivities

The 4D Flow MRI scan was performed using a 3T Ingenia scanner by Philips Healthcare. All three velocity directions were acquired, plus one acquisition with no encoding gradient to acquire the background phase. The scan parameters were set to TR = 8.9ms, TE = 4.5ms, FA = 8$^\circ$, *venc*=150 cm/s. The matrix size was [160, 160, 40] with a spatial resolution of [0.8mm, 0.8mm, 0.8mm] and a temporal resolution of 19 frames per cardiac cycle. The scan was accelerated with an acceleration factor of R=2. The scan used a 32-channel coil. During a pre-scan, the channels with the highest signal in the field-of-view were selected automatically by the scanner, leading to 15 coil measurements. Thus the total matrix size is [160, 160, 40, 4, 15, 19]. Examples of the measurements, both the k-space magnitude and different reconstructions, are shown in Fig. 5.

#### Forward problem setup

**Fig. 6:**
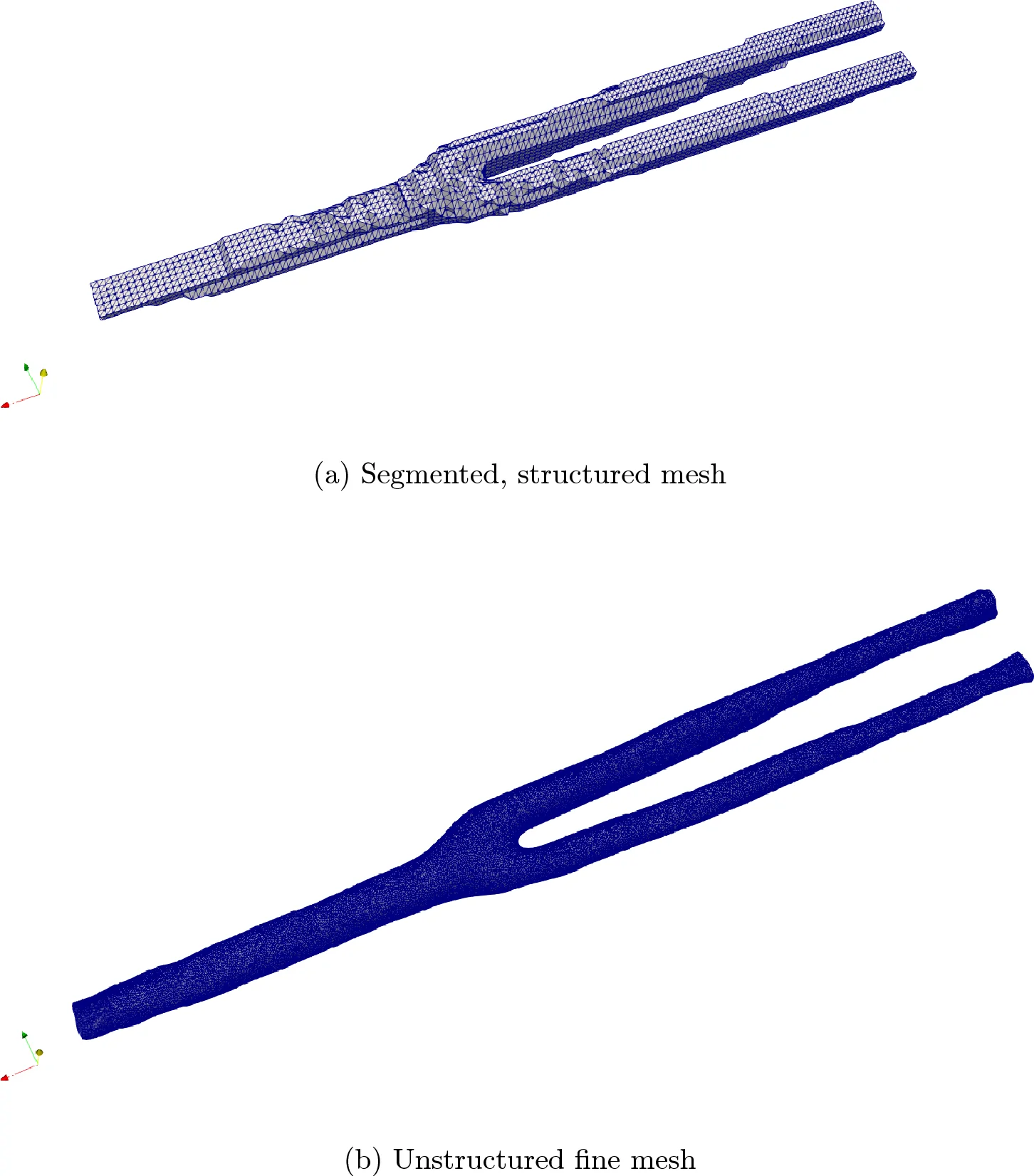
Meshes of the carotid phantom. The first mesh is a structured mesh created from a segmentation of the lumen, and the second mesh is an unstructured, finer mesh created from the first one. The second mesh was used for the forward problem

A structured mesh was created from a segmentation of the lumen, matching the spatial resolution of the scan. From this an expanded, higher-resolution mesh was created using Blender, Meshlab and gmsh. This mesh was used for the forward problem. The two meshes are depicted in Fig. 6.

We again model the blood flow with the incompressible Navier–Stokes equations, with the same choices for the physical parameters. The inflow boundary is modeled by a Dirichlet boundary condition with an inflow

21 $$\begin{aligned} \boldsymbol{u}_{in} = \boldsymbol{u}_{profile}U f(t) \end{aligned}$$

where $\boldsymbol{u}_{profile}$ is a flow profile provided by the solution of a Stokes problem in the domain, *U* is a constant amplitude, and *f*(*t*) defines the temporal profile as

22 $$\begin{aligned} f(t) = {\left\{ \begin{array}{ll} \sin (\frac{\pi t}{T}) \text { if } t \le \frac{3}{4} T \\ sin(\frac{3}{4}\pi )(1-t+\frac{3}{4}T)\exp ^{-(t-\frac{3}{4}T\beta }) \text { if } t > \frac{3}{4} T \end{array}\right. } \end{aligned}$$

with T=0.64, β=5. The temporal profile was determined empirically to match the shape of the flow rate in the phantom. The inflow profile $\boldsymbol{u}_{profile}$ is obtained by solving a Stokes problem in the domain with the same boundary conditions as the original problem, with a constant unitary Neumann load on the inlet and homogeneous Neumann conditions on the outlets. This provides a velocity profile that satisfies the incompressibility condition and the non-slip boundary condition on the wall. The profile is normalized to have a maximum value of 1, so that the velocity amplitude of the inflow is determined by *U*.

The two outlets were modeled with resistance boundary conditions

$$\begin{aligned} P_l&= R_{p, l} Q_l \text { on } \Gamma _l\\ Q_l&= \int _{\Gamma _l} \boldsymbol{u}\cdot \boldsymbol{n} dx \end{aligned}$$

As the flow division between the outlets is determined by the proportion of the resistances, we can fix $R_{p, 1} = 100$ at an arbitrary value.

#### Inverse problem setup

We estimate the inflow amplitude *U* and the resistance $R_{p, 2}$ of the boundary condition at the right outlet. The initial guess is U=100, $R_{p,2} = 100$, i.e. an equal division of flow between the outlets. The initial standard deviation was set to 0.5.

We are reparameterizing such that $\boldsymbol{\theta } = \boldsymbol{\theta }^0 \odot \boldsymbol{\nu }$ with $\boldsymbol{\theta }^0$ the initial guess for the parameters and the filter being applied to $\boldsymbol{\nu }$, with the initial value of $\boldsymbol{\nu } = \boldsymbol{1}$. This was done because the exponential reparameterization used before proved unstable in this case.

To extend the filter to multiple coils, each of the coil measurements was treated as a separate, independent measurement, resulting in 15 measurements per time step. The variance of the noise for each coil was estimated from the first measurement by computing the standard deviation of

23 $$\begin{aligned} \boldsymbol{Y}^0 - \boldsymbol{M}(t^0)\odot \exp \left( i\boldsymbol{\phi }_{back}(t^0))\right) \odot \boldsymbol{S} \end{aligned}$$

as before, which neglects potential spatial variation of the variance due to the sensitivity of the coils.

The observation operator requires measurements of the magnitude of the magnetization and the background phase. The background phase is reconstructed as

24 $$\begin{aligned} \boldsymbol{\phi _{back}} = \angle \mathcal {F}^{-1}(\boldsymbol{Y}_{back}) \end{aligned}$$

where $\boldsymbol{Y}_{back}$ is a zero-filled measurement acquired with R=2 and no encoding gradient, i.e. capturing only the background magnetization and no fluid velocity, and ∠ corresponds to the angle of the complex value.

For the magnitude, we consider two options. The first option is

25 $$\begin{aligned} \boldsymbol{M} = |\mathcal {F}^{-1}(\boldsymbol{Y})| \end{aligned}$$

for each velocity direction, where $\boldsymbol{Y}$ is a zero-filled measurement acquired with R=2 in that velocity direction. This however assumes that a highly sampled measurement is available. Therefore the other option is to use

26 $$\begin{aligned} \boldsymbol{M} = |\mathcal {F}^{-1}(\boldsymbol{Y}_{back})| \end{aligned}$$

for all velocity directions, using the same measurement as for $\phi _{back}$. This would only require a highly sampled measurement of one out of four encoding gradients.

**Fig. 7:**
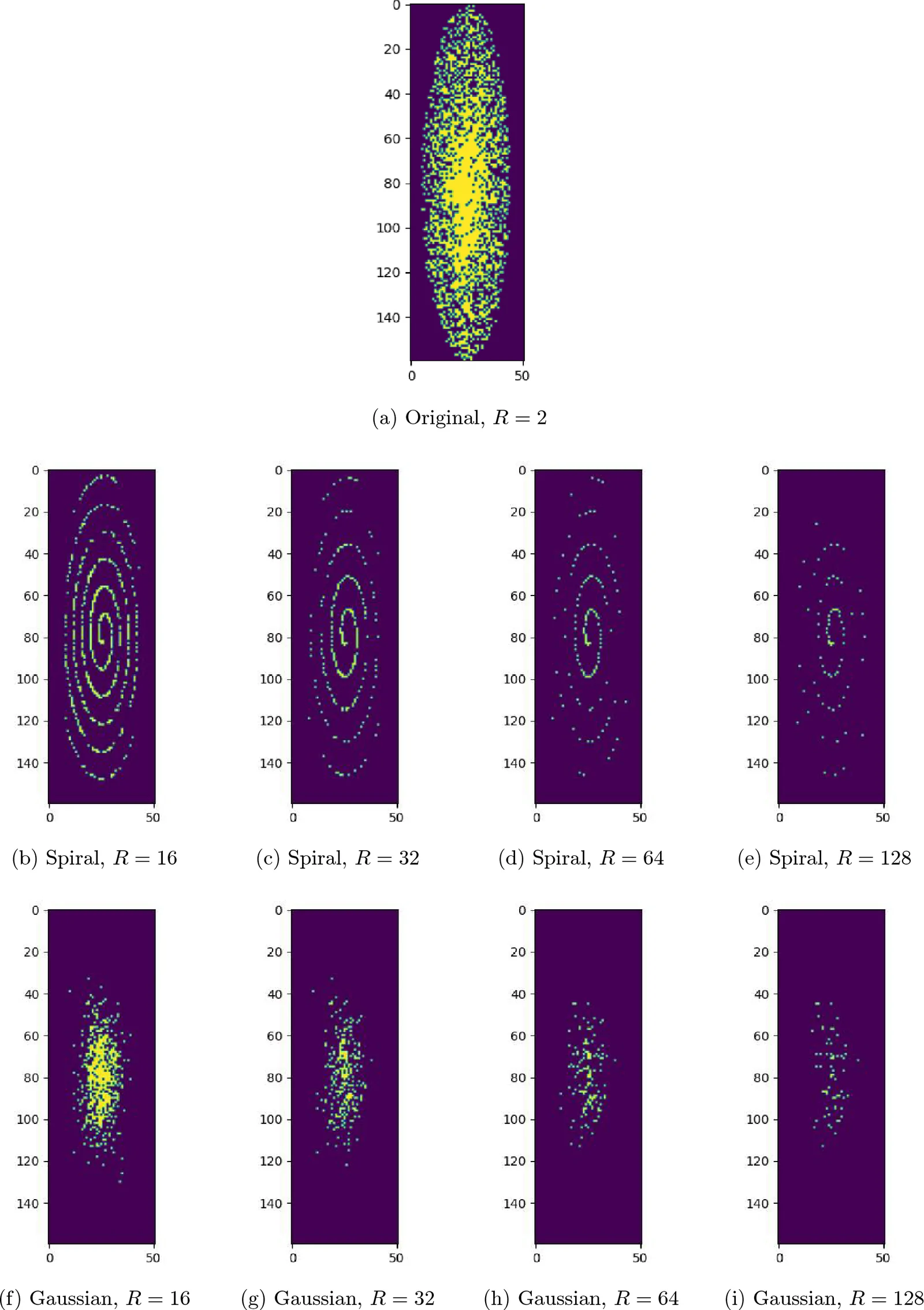
Sampling masks for the phantom data, taken at the $k_x = 0$ slice at time step 0

The original data with an acceleration factor of 2 were acquired with an incoherent pseudo-spiral sampling in the $k_y-k_z$ direction with a fully sampled $k_x$-direction. We further undersample this by applying masks with a Gaussian or spiral sampling pattern onto the sampling mask for R=2 to achieve higher acceleration factors R=16,32,64,128. In contrast to the masks for the synthetic data, this results in different masks for each time step and velocity direction. Examples of the resulting masks are shown in Fig. 7.

The measurements are assumed to occur every 0.053s ($\approx 1\,s/19$). For the inverse problem, the first two measurements (at 0.053s and 0.106s) are omitted, as negative velocity values in these measurements could interfere with the Kalman filter by leading to negative particle values, which would be unphysical for the forward simulation.

## Results

### Synthetic data

*Computational effort.* Parameter estimation from either velocity data or frequency data requires approximately the same CPU time (around 13 min on an AMD 7763 CPU), although the computation can be run in parallel to reduce wall time. Using velocity measurements requires slightly less memory, however, since no complex-valued data need to be saved (888.6 MB compared to 959 MB for the frequency-space data). Reconstructing the velocity data with BART requires an additional 1:30 min of CPU time and 660 MB of memory.

We compare the results of our method, which estimates parameters directly from frequency-space measurements, with those obtained by estimating parameters from velocity measurements reconstructed with compressed sensing using BART with an $\ell _1$-regularization in time. The regularization parameter was determined empirically by visual inspection for each combination of acceleration factor and mask and is listed in Table 3. For the estimation, we use the method presented in Garay et al. (2022) to take advantage of the known R=2 magnitude.

*Comparing the reconstructed flows.* In order to accurately reflect the different impact of the parameters on the flow, we compute the error in terms of the flow computed from the estimated parameters. Using the estimated parameters, we solve the forward problem 15 again with a time step of dt=0.001. The error is calculated as

27 $$\begin{aligned} e = \frac{||\boldsymbol{u}_{ref} - \boldsymbol{u}_{recon}||_{2}}{||\boldsymbol{u}_{ref}||_2} \end{aligned}$$

where $\boldsymbol{u}_{recon}$ and $\boldsymbol{u}_{ref}$ are vectors consisting of the velocity values for all components at each point in the geometry at each point in time stacked together, using the estimated parameter values for $\boldsymbol{u}_{recon}$ and the true parameter values for $\boldsymbol{u}_{ref}$.

**Table 3:** Regularization values λ used for the reconstruction with BART

Acc. Factor	Spiral	Gaussian
*R*1	0.0001	
*R*8	0.1	0.01
*R*16	1.0	0.01
*R*32	10.0	1.0

**Fig. 8:**
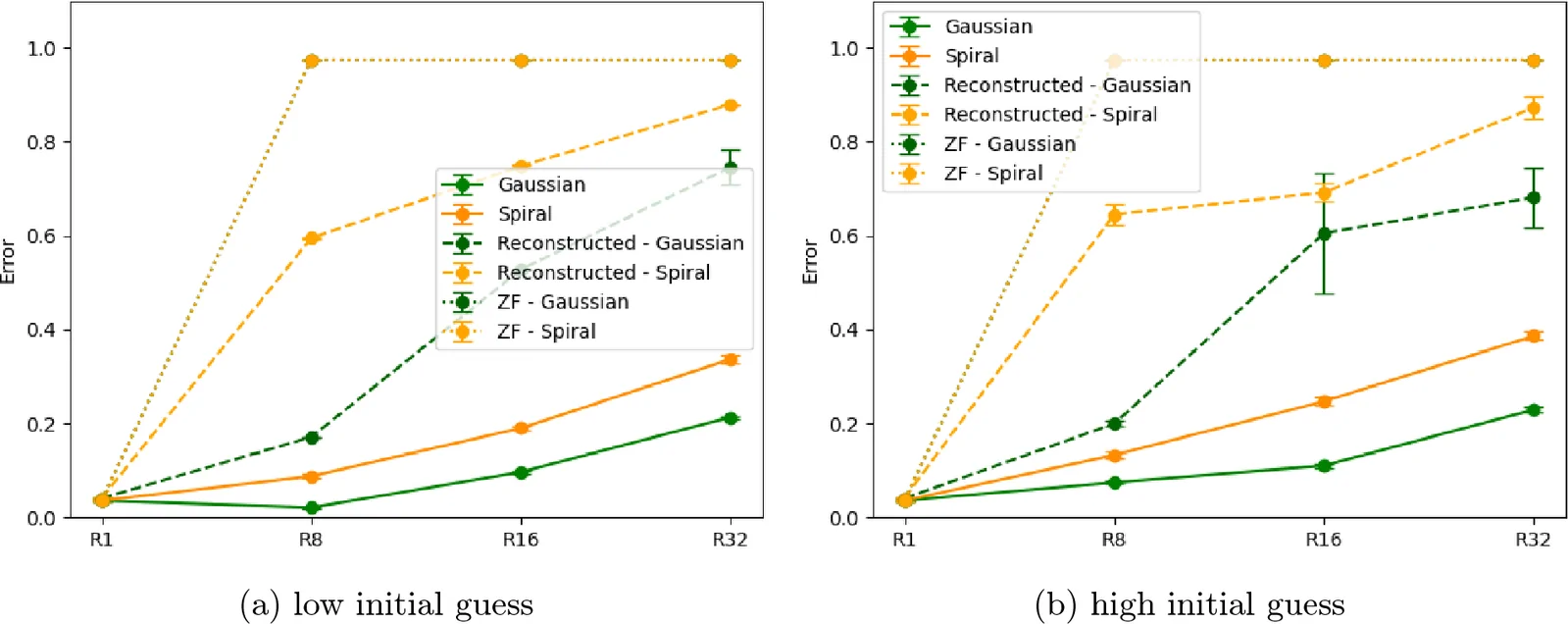
Error values for different acceleration factors $R=\{1,8,16,32\}$ and different masks (Gaussian and spiral). Dotted lines are the error values of the flow reconstructed from the parameters estimated by the inverse problem using zero-filled measurements, dashed lines for the flow reconstructed from the inverse problem from BART velocities, solid lines are from the inverse problem in k-space. Low initial guess on the left, high initial guess on the right. The bars indicate the standard deviation of the error

The error values for all acceleration factors for the low and high initial guesses are depicted in Fig. 8 for our method, for parameter estimation using velocities reconstructed with BART, and for the error values of the velocities reconstructed with BART itself (without first estimating the parameters). In the latter case, $u_{ref}$ was interpolated onto the coarser mesh to match the resolution of the reconstructed velocities. In all cases, estimating the parameters and then reconstructing the flow achieves better results than using zero-filled velocity measurements. For the k-space cost function, the errors of both masks are very close to the error with fully sampled data for R=8 and increase for R=16 and R=32. The Gaussian mask achieves lower error values for all three acceleration factors.

Considering the velocity measurements from data reconstructed using BART, it can be seen that the Gaussian mask performs considerably better than the spiral mask, especially for R=8. This matches the expectation, as a pseudo-random mask leads to incoherent artifacts that can be more easily excluded with a temporal regularizer. Nonetheless, the error increases drastically for R=16 and R=32. The spiral mask shows a strong increase in error already for R=8 and remains high at higher subsampling rates.

Using frequency measurements directly outperforms using the reconstructed velocity measurements for all acceleration factors except for the fully sampled case.

Both the high and low initial guesses show the same pattern. As such, from here on, we limit ourselves to showing results for the low initial guess, as those for the high initial guess provide no additional information.

**Fig. 9:**
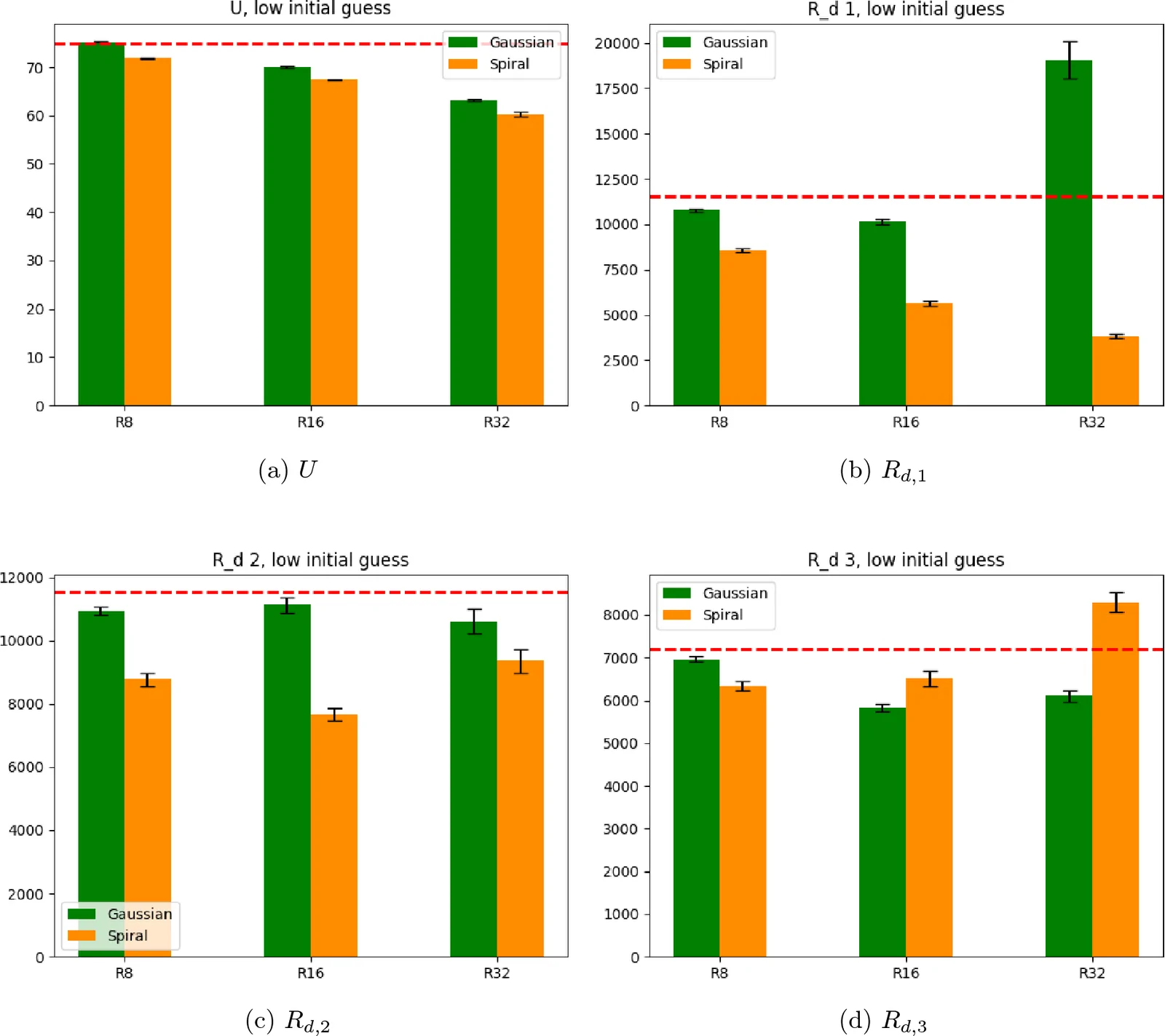
Estimated values of individual parameters $R_{d,1},R_{d,2},R_{d,3}$ in the Windkessel model and peak inlet velocity *U*, for acceleration factors $R=\{8,16,32\}$, as well as the sampling mask (Gaussian and spiral). The dashed line indicates the true value from which the measurements were generated

*Comparing the parameter values.* Comparing the estimated values of the individual parameters in Fig. 9, it is apparent that the accuracy of the estimation differs by parameter. The inflow *U* is estimated relatively accurately by all masks, with only a small decrease in accuracy as *R* increases. In comparison, the distal resistance of the first Windkessel outlet ($R_{d,1}$) is considerably underestimated by the spiral mask, and this underestimation increases with *R*, while the Gaussian mask estimates it well for R=8 and R=16 but severely overestimates it for R=32. $R_{d,2}$ and $R_{d,3}$ show the same pattern, with the spiral mask underestimating the values relative to the Gaussian mask, though more severely for $R_{d,2}$ than for $R_{d,3}$. For R=32, the spiral mask slightly overestimates the value of $R_{d,3}$.

**Fig. 10:**
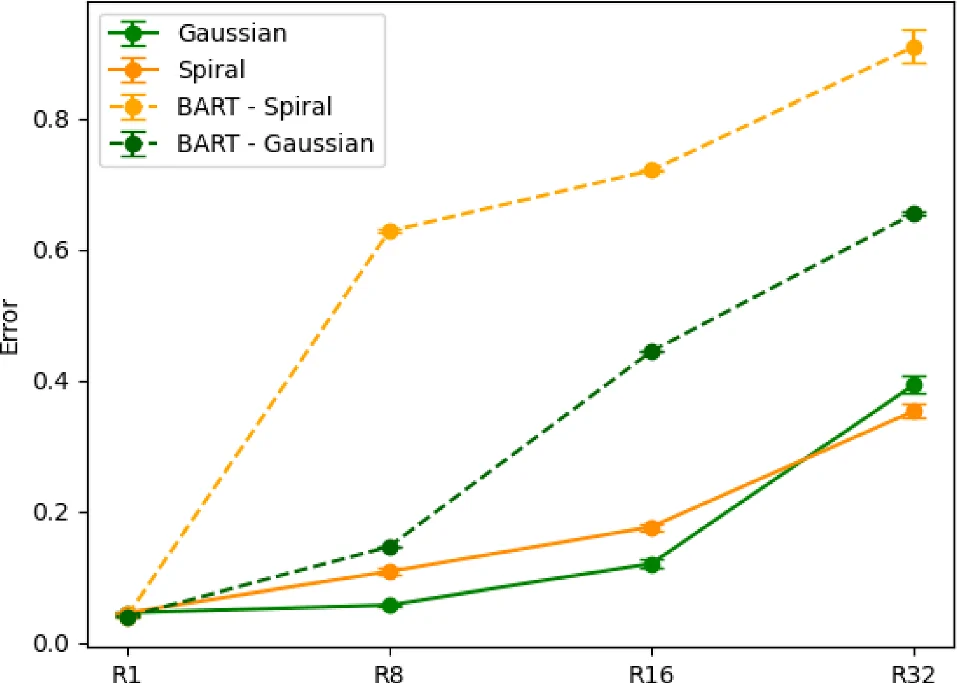
Error values in the computed velocities from the estimated parameters for different acceleration factors, for Gaussian and spiral masks, using only the *z*-component of the velocity. “BART" means that the parameters were estimated from the velocity measurements reconstructed with BART. The bars indicate the standard deviation of the error

*Using only a single velocity component.* We also consider the case where measurements are available for only one of the velocity components, in this case the *z*-component, which corresponds to the foot-head direction in this setup. Figure 10 shows the error values for this case. They follow the same patterns as when using all velocity components and are only slightly higher. Here, the difference in error values between the spiral and Gaussian masks is smaller for R=8 and R=16, and the spiral mask shows a flatter error curve, whereas the error for the Gaussian mask increases at R=32. Again, using frequency measurements yields lower error values than using BART measurements for all subsampled data.

**Fig. 11:**
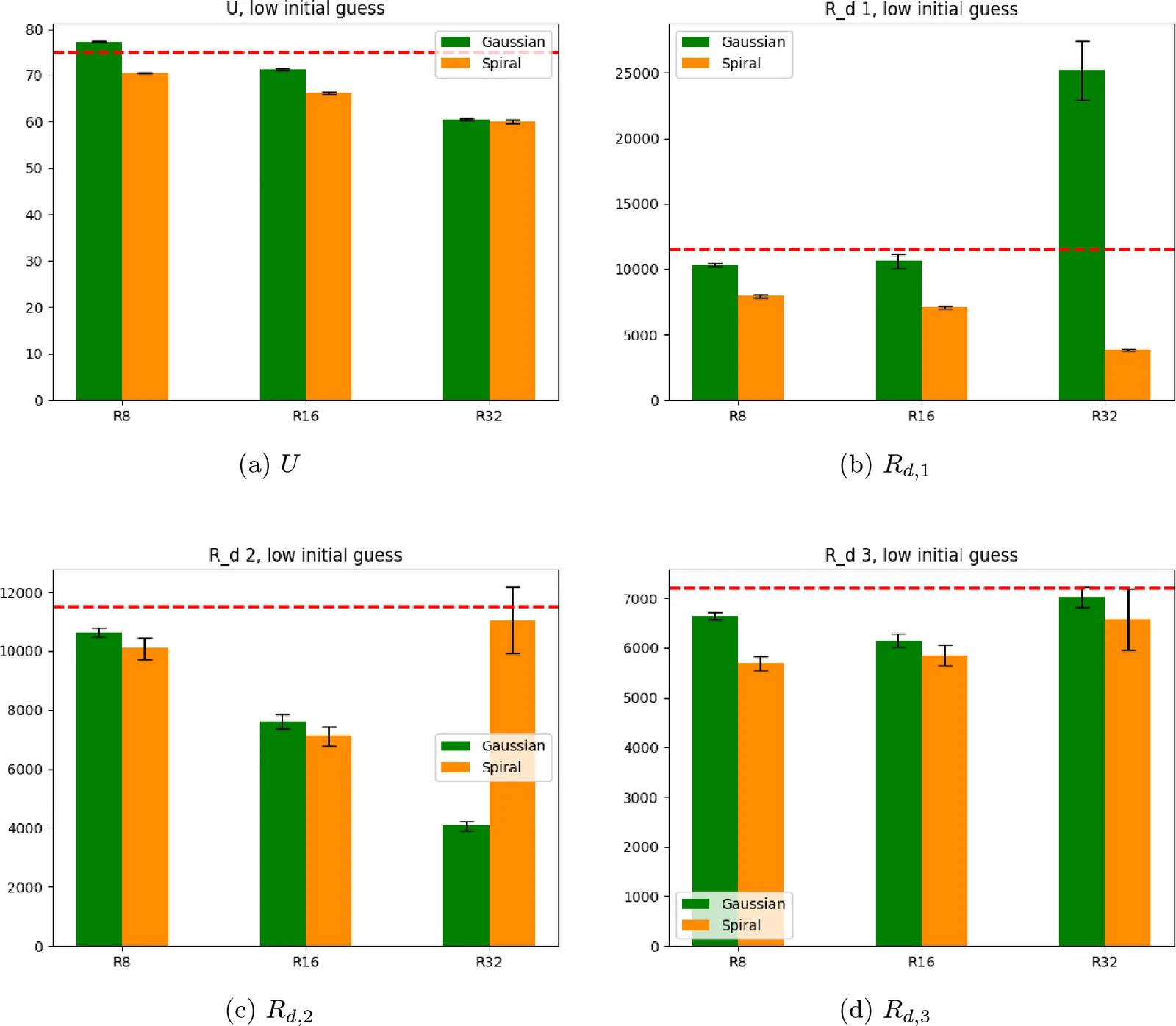
Estimated values of individual parameters $R_{d,1},R_{d,2},R_{d,3}$ in the Windkessel model and peak inlet velocity *U*, for acceleration factors R={8,16,32}, as well as the sampling mask (Gaussian and spiral), but measuring only the z-component of the velocity. The dashed line indicates the true value from which the measurements were generated

When considering each parameter separately in Fig. 11, the same patterns and similar values persist as for all velocity components. The exception is the Gaussian mask for $R_{d,2}$, which now significantly underestimates the values, unlike the spiral mask.

*Robustness to the choice of* *venc* **.**

As described previously, a low *venc* leads to a high signal-to-noise ratio but may also cause aliasing artifacts if the actual maximum velocity exceeds *venc*. The cost function used in Garay et al. (2022) addresses this by distinguishing the actual velocity from the wrapped velocities when the physical parameters affect the velocity at several voxels simultaneously. In the present work, the cost function is similar to that in Garay et al. (2022) but includes an additional Fourier transform and thus also accounts for aliasing compensation.

**Fig. 12:**
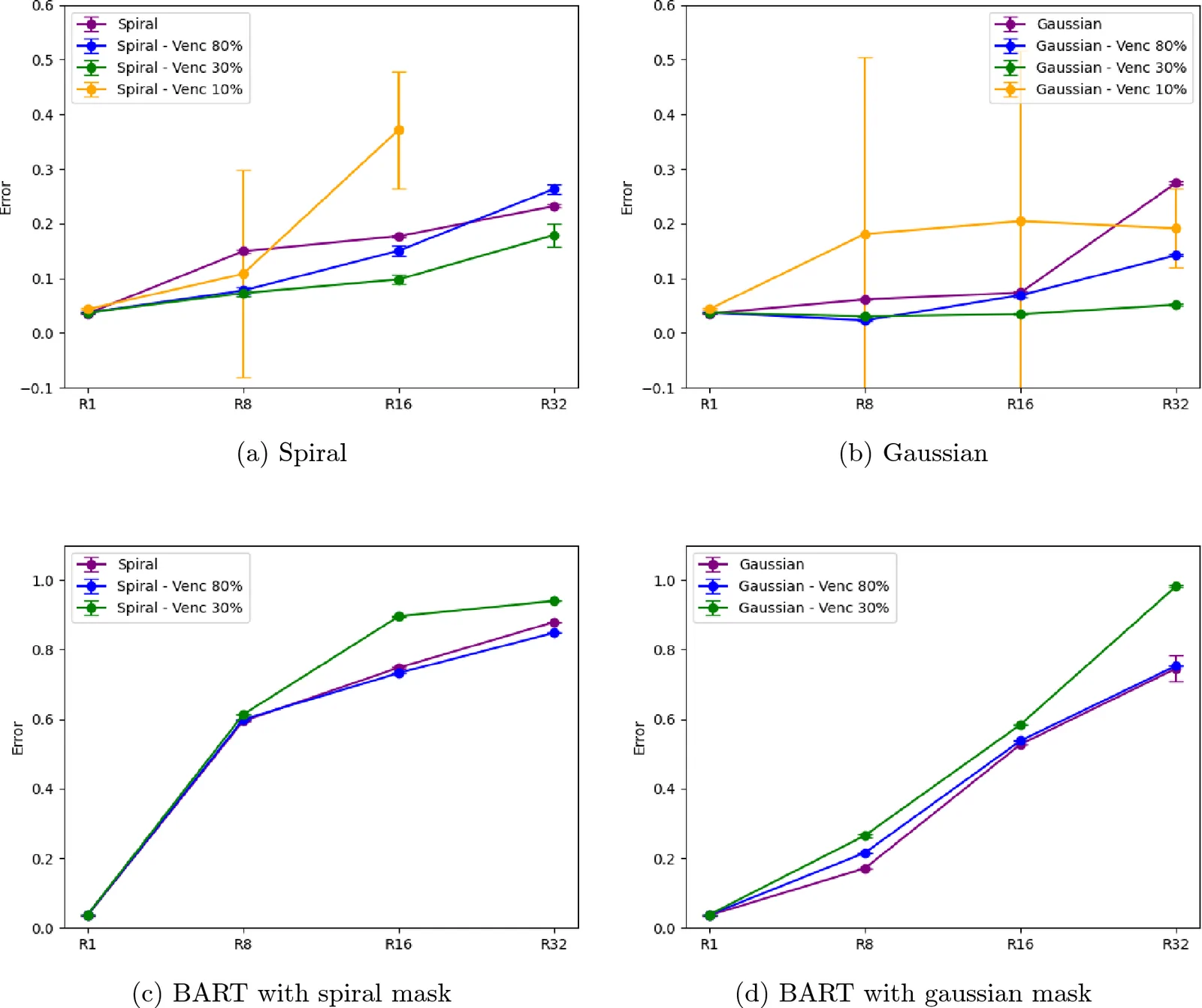
Error values for different *venc* values, corresponding to 100%, 80%, 30%, and 10% of the maximum velocity. Spiral mask on the left and Gaussian mask on the right. The value for the spiral mask at R=32 and $venc = 10\% V_{\max }$ is excluded because the inverse problem failed to converge. For the BART reconstructions, $venc = 10\% V_{\max }$ was not considered since it did not converge either

To investigate the robustness of our method to choosing *venc* values lower than the maximum velocity, thereby exploiting the higher sensitivity of the signal to velocity, we compare three additional *venc* values corresponding to 80%, 30%, and 10% of the maximum velocity. The results are shown in Fig. 12.

The results improve considerably as *venc* decreases to 80% and 30%, especially at higher acceleration factors. However, the smallest *venc* of 10% leads to increased errors and standard deviations, and the Kalman filter even fails to provide results for the spiral mask at R=32. The results for both masks behave similarly as *venc* decreases, indicating that this response is inherent to the inverse problem formulation – as shown in Garay et al. (2022) – rather than to the choice of mask.

By comparison, for the BART reconstructions, the error remains similar for $venc = 80\% V_{max}$ and then increases significantly for $venc = 30\% V_{max}$. This behavior can be explained by the fact that the reconstructed velocities were already unwrapped before parameter estimation, as described in Garay et al. (2022).

*Using an estimated magnitude of the magnetization.* In the previous results, we used a magnitude reconstructed from k-space subsampled with R=2. To assess the accuracy of this substitution, we now compare it with the case of perfect knowledge of the magnitude. We also compare it with using a constant magnitude value of 0.5 to model having little to no knowledge of the magnitude.

**Fig. 13:**
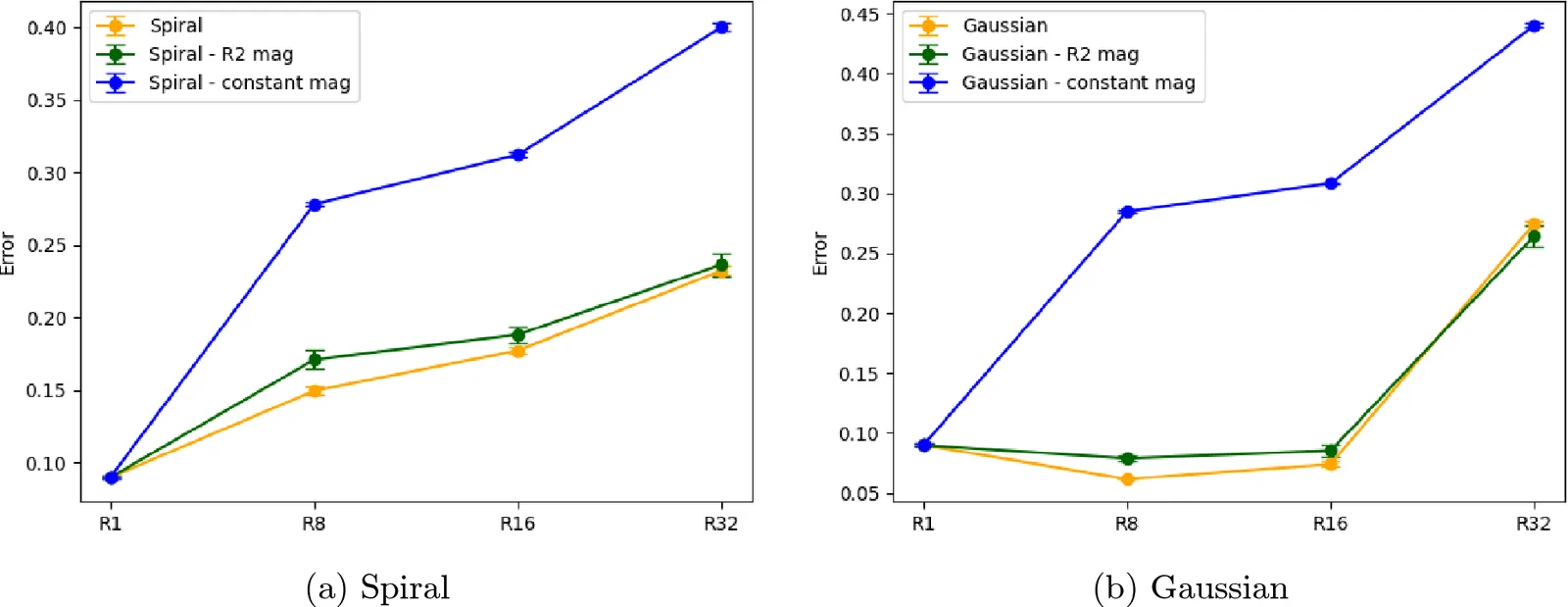
Error values for the computed velocities from estimated parameters for different estimations of the magnitude, for $R=\{1,8,16,32\}$, and Gaussian and spiral masks. The bars indicate the standard deviation of the error

As can be seen in Fig. 13, using the magnitude reconstructed from frequency data with an acceleration factor of R=2 yields results that are very close to those obtained using the perfect magnitude. On the other hand, using a constant magnitude leads to a significant increase in error that appears to remain constant across the different acceleration factors. In this case, there are also fewer differences between the two masks. Nonetheless, for higher acceleration factors, even with a constant magnitude, the error is lower than that obtained with velocity measurements reconstructed using BART.

**Fig. 14:**
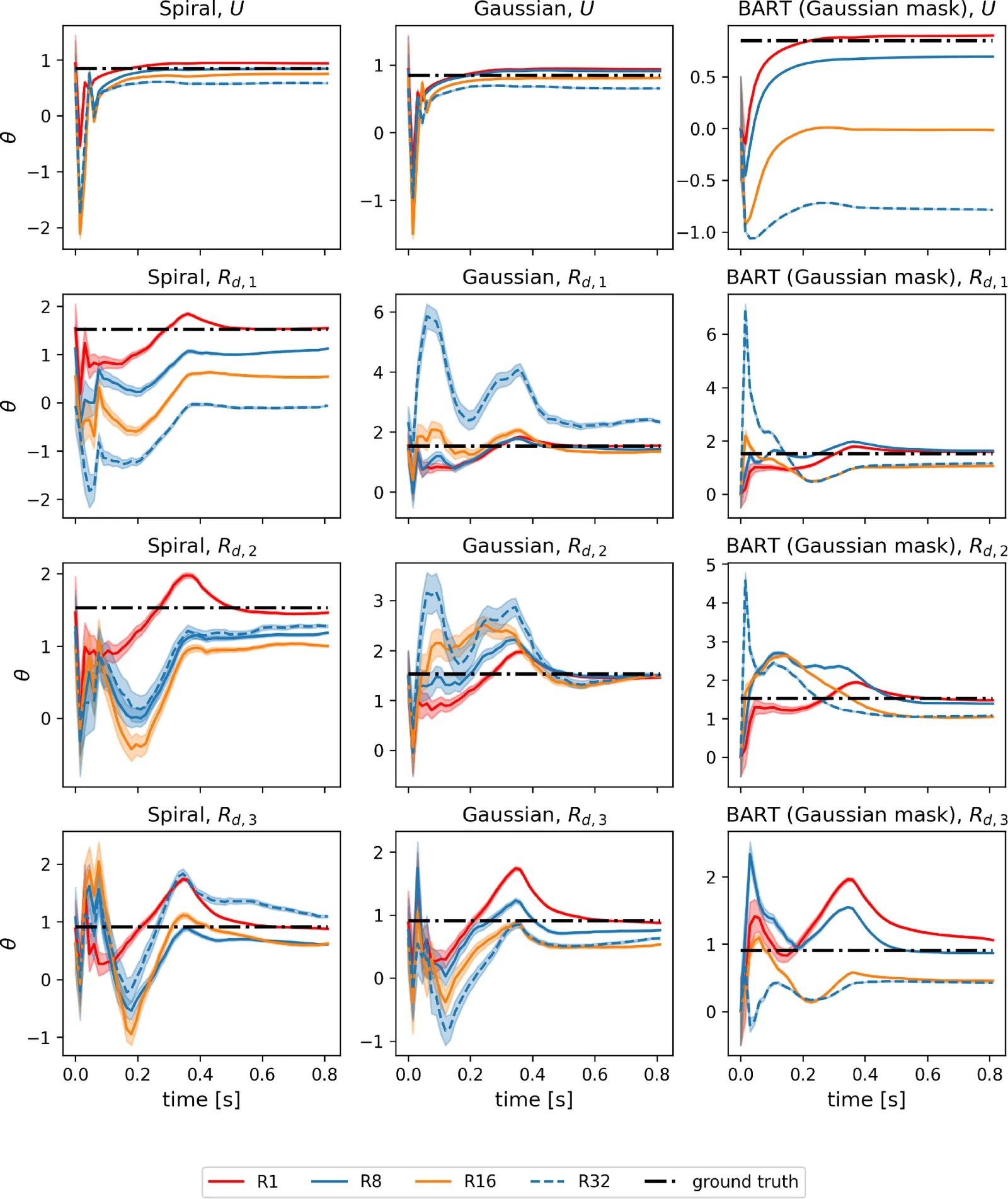
Evolution of the estimated parameters $U,R_{d,1},R_{d,2},R_{d,3}$ over the time of the ROUKF simulation for a sample noise realization. The dashed line indicates the true value from which the measurements were generated. The results are shown for acceleration factors $R=\{1,8,16,32\}$ and for the spiral and Gaussian masks for our method, as well as for the BART reconstruction with the Gaussian mask. The shaded areas indicate the parameter standard deviations output by ROUKF

*Parameter curves of the Kalman filter.* We also provide examples of the ROUKF parameter curves in Fig. 14, which show the evolution of the estimated parameters over the course of the simulation. The shaded areas indicate the parameter variances. For each mask using our new method, these variances are higher at the end of the simulation for larger *R*, since less data is available. This effect is not observed for the BART-reconstructed velocities, for which the variances decrease similarly across all acceleration factors. Moreover, these variances are much smaller than those obtained with the frequency-space-based method, indicating higher confidence in the estimated parameters. This higher confidence stems from the fact that, due to the reconstruction, the measurements contain more data than in k-space (they now have as many voxels as the image space, rather than only the actually measured voxels). However, since these data originate from the same subsampled measurements, they do not actually contain more information, leading to a misleading quantification of the uncertainty in the inverse problem.

Furthermore, for low *R*, the curves qualitatively follow the shape of the corresponding curve for the fully sampled data, but at higher acceleration factors, the choice of mask affects the evolution of the parameter estimates not only quantitatively but also qualitatively. The curves produced by our method also differ considerably from those obtained using BART, which develop erratic spikes in either direction as the acceleration factor increases. By comparison, the curves for each mask remain more similar to one another as *R* increases.

It can also be seen that, in all cases, the estimated parameters are mostly constant after roughly t=0.5, and the parameter variances do not decrease considerably after that. This indicates that, for both frequency-space data and reconstructed data, the relevant information is located entirely within systole.

In summary, for the synthetic data:

- solving the inverse problem in k-space significantly reduces the error;
- there are notable qualitative and quantitative differences in the parameter estimates between the different masks;
- the method is robust to different values of *venc* and to the use of estimated magnitudes and background phases.

### Phantom data

**Fig. 15:**
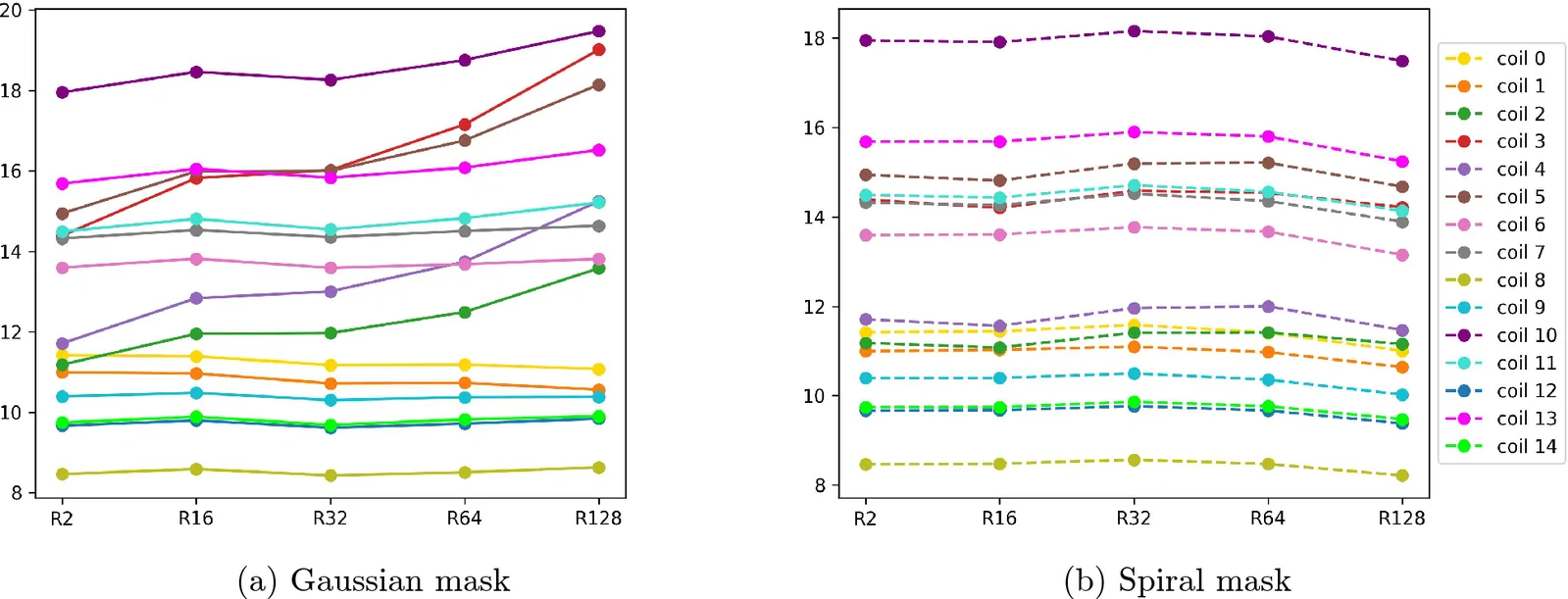
Estimation of the standard deviation of the signal noise for all 15 coils for the carotid phantom data set, for acceleration factors $R=\{2,16,32,64,128\}$ for the Gaussian and spiral masks

First, we consider the quality of the estimated standard deviation of the noise for the phantom data. The results for each mask and acceleration factor are shown in Fig. 15. While the estimated noise for the spiral mask remains nearly constant across acceleration factors, the estimates for the Gaussian mask show large variations for some coils. This is likely due to the spatial variation in signal strength among some coils and to the fact that the Gaussian mask does not uniformly cover the space. Nonetheless, this approach provides a good approximation of the standard deviation of the noise even at high acceleration factors.

For comparing the reconstructed flows, we are using the same error metric (27) as in Sect. 4.1, with $\boldsymbol{u}_{ref}$ the solution of a forward problem with the parameters estimated from R=2.

**Fig. 16:**
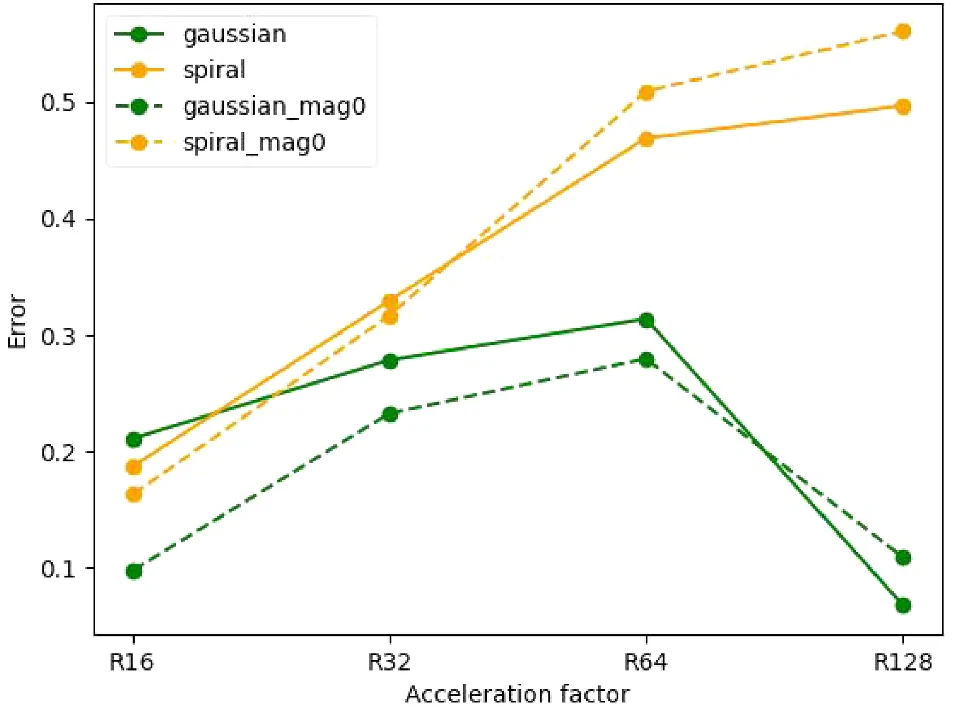
Error values for the MRI phantom data in the computed velocity for acceleration factors $R=\{16,32,64,128\}$ for both spiral and Gaussian masks. The "mag0" labelled results use the magnitude from the acquisition without a velocity encoding gradient

The error values can be seen in Fig. 16, where a considerable difference in the behavior of the two masks is noticeable. Both achieve similar results for an acceleration factor of R=16, but the error increases more for the spiral mask than for the Gaussian mask. The Gaussian mask also shows a decrease in error from R=64 to R=128, which may be the result of differences in the noise estimation or simply an outlier. Both choices of magnitude in the observation show similar results, implying robustness when using a magnitude from a different velocity direction.

**Fig. 17:**
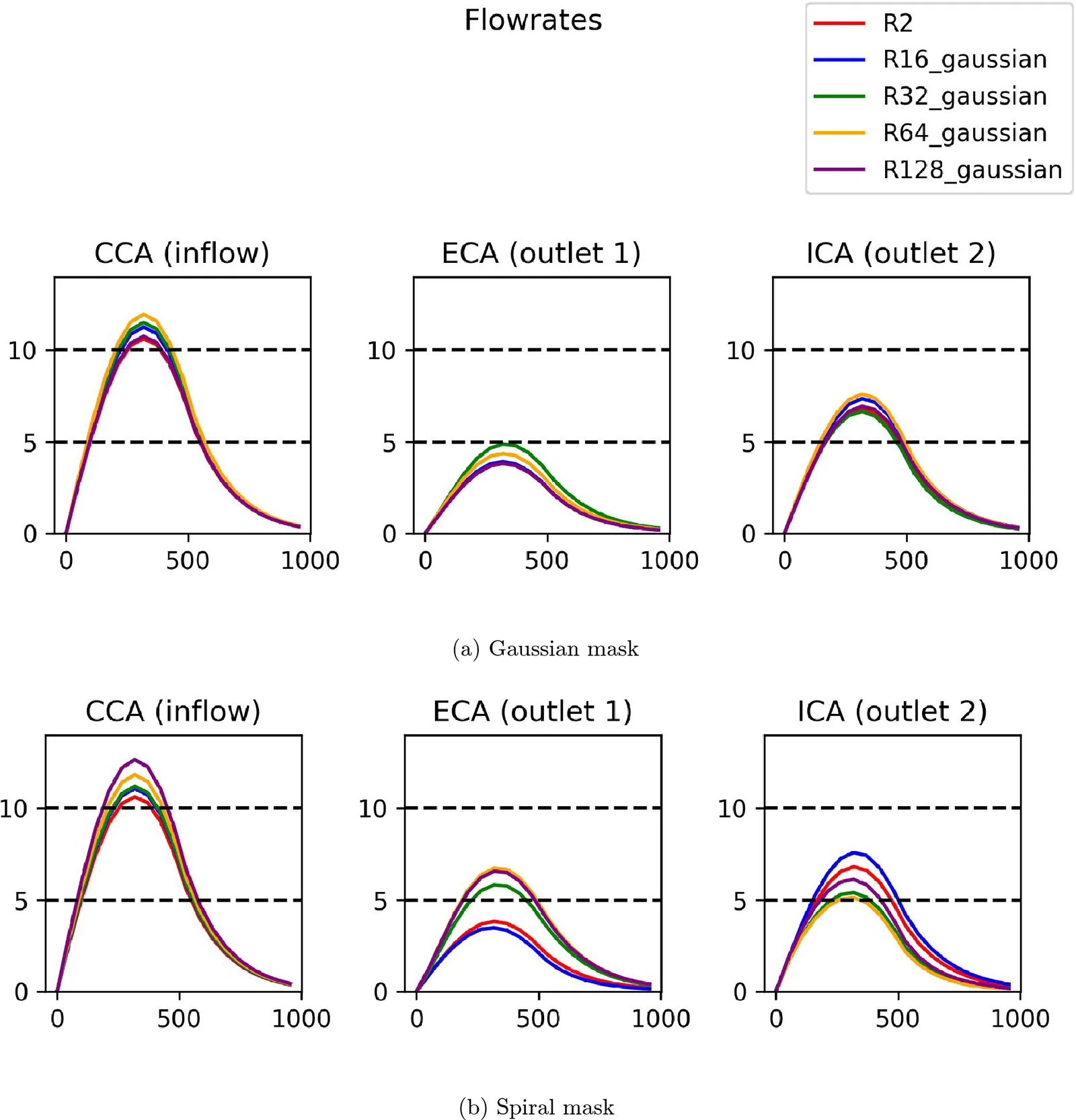
Flow rates at the inlet and each of the outlets of the forward simulations based on the estimated inflow and resistance parameters for the phantom data, for acceleration factors $R=\{16,32,64,128\}$ and for the Gaussian and spiral masks

**Fig. 18:**
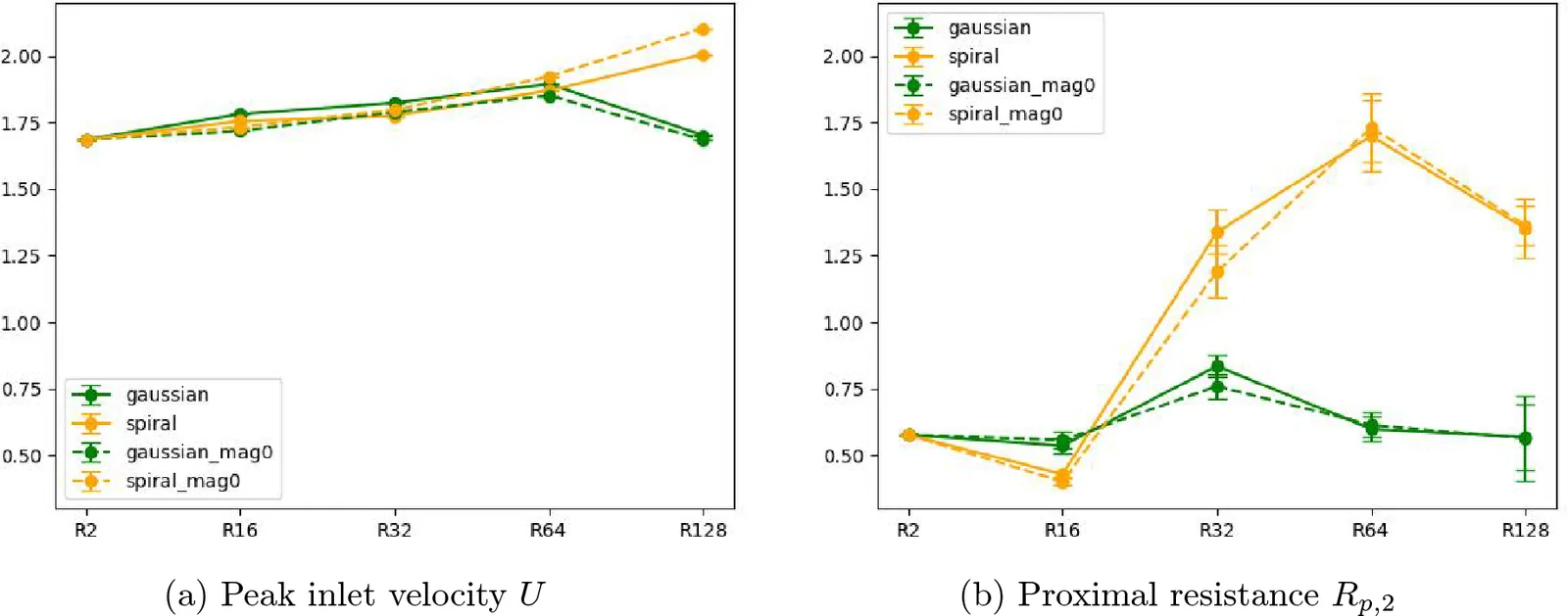
Estimated parameter values in the phantom MRI dataset for acceleration factors $R=\{2,,16,32,64,128\}$ for both spiral and Gaussian masks. Error bars depict the estimated parameter variance by the ROUKF, multiplied by a factor of 1000 for visibility. The "mag0" labelled results use the magnitude from the acquisition without a velocity encoding gradient

In Fig. 17, we show the flow rates of the reconstructed flows at the inlet and at each outlet. Both masks tend to overestimate the inflow as the acceleration factor *R* increases. For the Gaussian mask, the flow split between the two outlets remains fairly consistent as *R* increases, whereas the spiral mask no longer accurately depicts the flow split from R=32 onward. This can also be observed in the estimated parameter values shown in Fig. 18. Both masks show a very consistent estimate of the inflow amplitude *U*, with differences only for R=128, but the estimate of the resistance boundary condition $R_{p,2}$ shows larger changes across acceleration factors. The spiral mask, in particular, overestimates $R_{p,2}$ at higher acceleration factors, leading to a reduced distinction between the outlets in the flow rates. A potential explanation for the drop in the errors of the estimated parameters at the largest acceleration factor is that, for higher acceleration factors, the exclusion of measurements that may contain more noise leads to a more accurate parameter estimation.

**Fig. 19:**
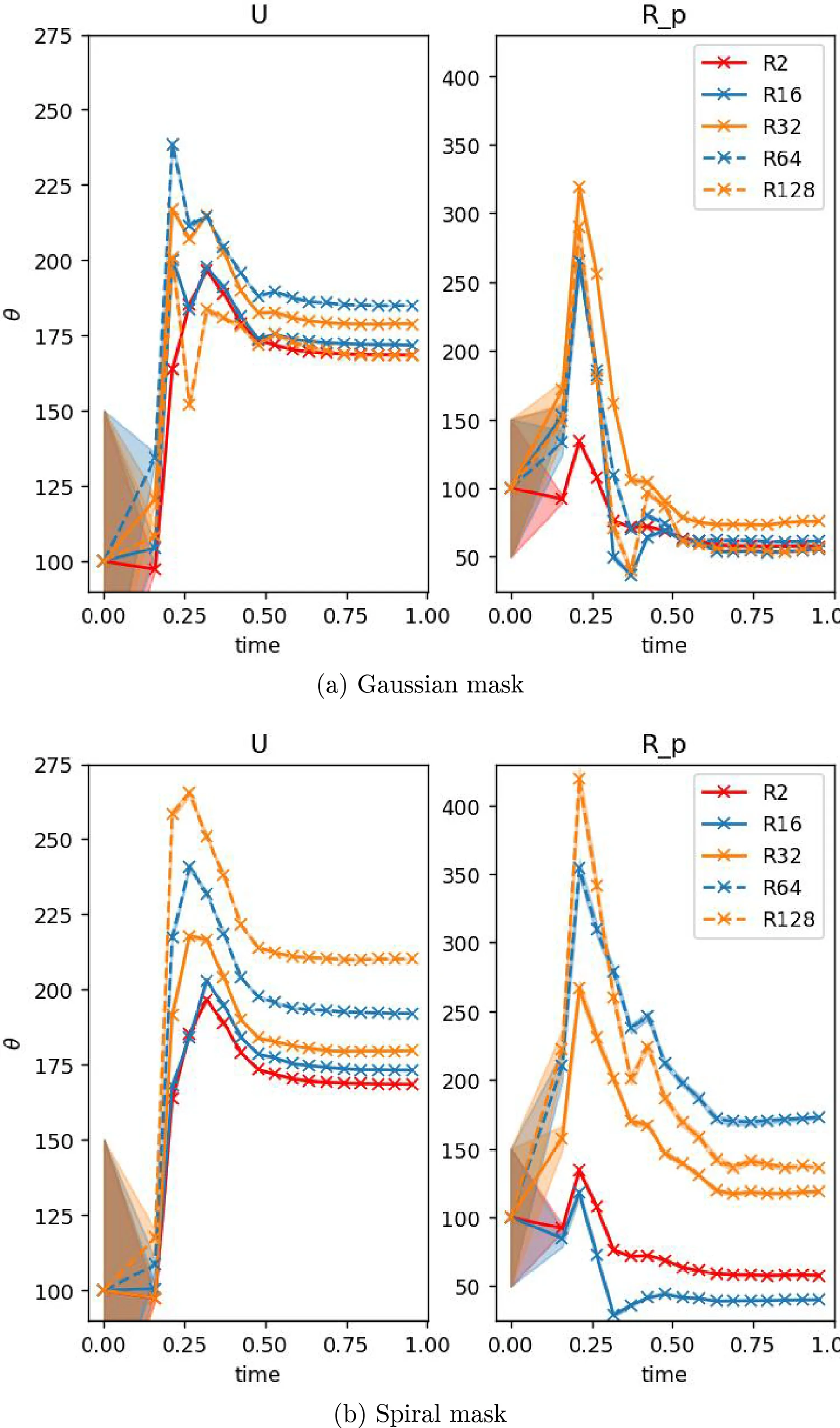
Evolution of the estimated resistance $R_p$ and inlet velocity *U* parameters in the MRI phantom dataset over the ROUKF simulation time from both spiral and Gaussian masks for acceleration factors $R=\{2,16,32,128\}$. Crosses mark times where corrections are made according to the available measurements. The shaded areas indicate the parameter standard deviations output by ROUKF

Examples of the ROUKF parameter curves are shown in Fig. 19. The curves for the Gaussian and spiral masks have similar shapes, with some qualitative differences. The acceleration factor appears to have a greater effect on the parameter evolution curves for the spiral mask than for the Gaussian mask. The parameter variance also decreases drastically after the first measurement and remains very small afterward, despite continuing changes in the parameter values.

In summary, for the phantom data:

- the method successfully estimates the parameters with time-dependent, estimated magnitudes, background phases, and noise levels;
- using the Gaussian mask, the estimation is reasonably accurate even for very high acceleration factors (R=128);
- the results differ both quantitatively and qualitatively between the two masks, with the Spiral mask failing to distinguish between the two outlets at higher acceleration factors.

## Discussion

### Summary of results

This work presents a new formulation of the inverse problem of parameter estimation in fluid flow problems using undersampled frequency-space MRI data and demonstrates it using a Reduced-Order Unscented Kalman Filter.

The numerical results are consistent across the synthetic and phantom datasets. In both cases, our approach robustly estimates the boundary conditions of the flow, even at high undersampling rates and low *venc* values, and outperforms the conventional approach of reconstructing velocity measurements from undersampled data through Compressed Sensing.

The results also show significant differences between the two sampling patterns studied, with qualitatively and quantitatively different outcomes for the inverse problem. The sampling patterns appear to differ in how accurately they estimate different parameters, indicating that the choice of sampling pattern may depend on which parameter is of greatest interest.

### Related work

In Müller et al. (2018), the authors used Fourier-transformed time-varying measurements from a single spatial point to estimate parameters of a one-dimensional fluid flow model, but neither the model nor the inverse problem exploited the potential of using velocity data from the entire spatial domain in the frequency domain. Additionally, Zhao et al. (2016) used an Unscented Kalman Filter with undersampled frequency-space data to retrieve T2 mappings for MRI brain scans. However, that approach relies on the assumption that the phase is both known and constant, and therefore uses only the magnitude of the k-space images while estimating the state rather than a set of parameters. Additionally, the authors only experimented with relatively small acceleration factors (up to a factor of 8).

It is worth noting that the presented approach is very different from physics-informed data regularization approaches; see, e.g., Fathi et al. (2020); Arzani et al. (2021); Zhang et al. (2022) for several reasons. First, data regularization approaches typically operate on reconstructed velocity data, whereas our approach directly uses highly undersampled k-space measurements. Moreover, data regularization approaches rely on weighting the data fidelity term and the (physics-based) regularization term, which can be challenging to tune and may not always lead to optimal results. In contrast, our PDE-constrained inverse approach – exemplified here within a Kalman filter framework – is regularization-free (the effect of Tikhonov regularization is removed through a few fixed-point iterations of the Kalman filter). Finally, we aim to estimate model parameters in order to obtain a digital twin, which is a different objective from data regularization.

### Limitations and future work

A limitation of the method is that it requires an accurate estimate of the magnitude and the background phase of the magnetization. In practice, this can be obtained by keeping the background image highly sampled, as we have shown in the phantom dataset, while highly undersampling the remaining three flow directions.

Our current approach is a proof of concept, and further validation on in-vivo data is necessary to fully assess its clinical utility. In particular, the simulated measurements do not fully capture the complexity of in-vivo data, and future work should focus on incorporating more realistic magnitude information by, for instance, embedding the (simulated) phase data in a real anatomical image and transferring magnitude information transferred into the model.

Another limitation of this paper is the absence of subject data, which would introduce additional complexities such as respiratory motion of the vessels due to breathing and the potentially large size of patient-specific meshes. Additionally, the noise present in the reconstructed magnitude and background phase is currently not considered by the Kalman filter. We plan to address this aspect in future research.

## Conclusion

We have proposed a new formulation of the inverse problem for parameter estimation in fluid flow problems using undersampled frequency-space MRI data and demonstrated it using a Reduced-Order Unscented Kalman Filter. This method outperforms parameter estimation based on velocity data reconstructed with Compressed Sensing, especially at high undersampling rates and for different *venc* values.

The choice of subsampling mask has a strong influence on the estimation of some parameters. Future work could therefore address the challenge of finding optimal sampling masks for certain parameters.

## Figures and Tables

**Algorithm 1:**
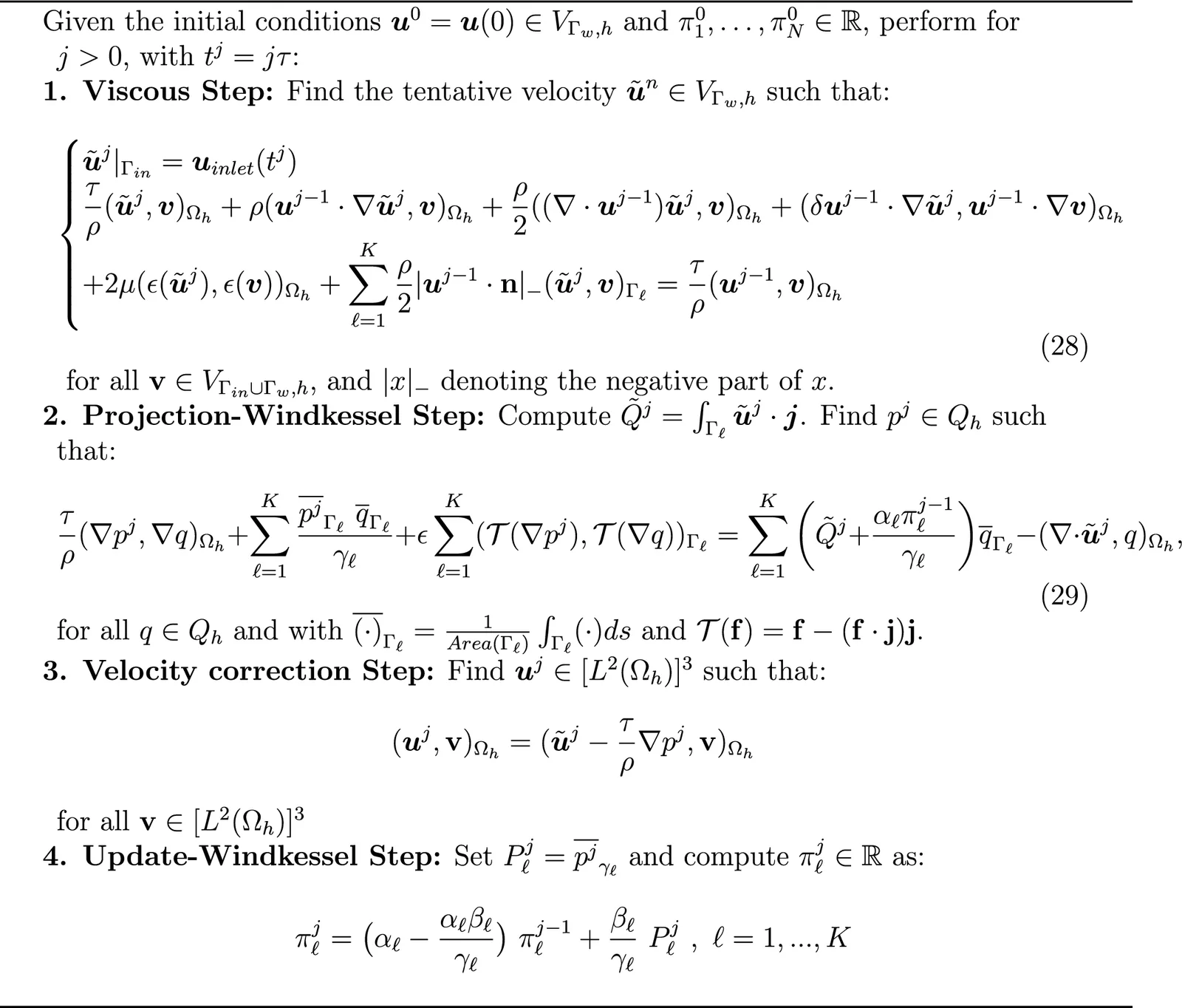
Fractional step algorithm with a modified semi-implicit Windkessel model coupling

## Data Availability

The data generated for this study was created using proprietary code that is not publicly available due to university policies. The data is available upon request and with permission. The experimental phantom data is not publicly available, but may be available upon request and permission by Pim van Ooij.

## A Numerical solution method of the forward problem

Here we detail the algorithm used to solve the incompressible Navier–Stokes equation with Windkessel boundary conditions for the forward problem.

## ROUKF algorithm

Here we detail the ROUKF algorithm adapted from Moireau and Chapelle (2011). Let us first consider the notation $[\boldsymbol{Z}^{(*)}]$ as the matrix with the column-wise collection of vectors $\boldsymbol{Z}_{(1)},\boldsymbol{Z}_{(2)},\dots$.

Define the *canonical sigma-points* $\boldsymbol{I}_{(1)},\dots ,\boldsymbol{I}_{(2p)} \in \mathbb {R}^p$ such that

30 $$\begin{aligned} \boldsymbol{I}_{(i)} = {\left\{ \begin{array}{ll} \sqrt{p}\boldsymbol{e}_i, & \text {for } 1\le i \le p\\ -\sqrt{p}\boldsymbol{e}_{i-p}, & \text {for } p+1 \le i \le 2p \end{array}\right. } \end{aligned}$$

where the vectors $\boldsymbol{e}_i$ form the canonical base of of $\mathbb {R}^p$. Moreover, define the weight $\alpha = \frac{1}{2p}$.

We denote by $\boldsymbol{\hat{X}}^n_-,\boldsymbol{\hat{X}}^n_+ \in \mathbb {R}^r$ a priori (model prediction) and a posteriori (corrected by observations) estimates of the true state $\boldsymbol{X}^n\in \mathbb {R}^r$. In the semi-implicit coupled 3D-0D fractional step Algorithm 1, the state consists in the velocity field $\boldsymbol{u}^n$ and the Windkessel pressures $\pi ^n_\ell$. Estimates of all unknown parameters are summarized by the corresponding a priori and a posteriori vectors $\boldsymbol{\hat{\theta }}^n_-,\boldsymbol{\hat{\theta }}^n_+\in \mathbb {R}^p$. The discretized forward model is written as $\boldsymbol{X}^n = \mathcal {A}^n(\boldsymbol{X}^{n-1},\boldsymbol{\theta }^{n-1})$, $\mathcal {A}^n$ denoting the model operator.

For given values of the initial condition $\boldsymbol{\hat{X}}^0_+ = \boldsymbol{X}^0 \in \mathbb {R}^r$, the initial expected value of the parameters $\boldsymbol{\hat{\theta }}^0_+ = \boldsymbol{\theta }^0 \in \mathbb {R}^p$ and its covariance matrix $\boldsymbol{P}^0$, perform

- *Initialization:* initialize the sensitivities as
  31a $$\begin{aligned} \boldsymbol{L}_{\theta }^{0}= & \sqrt{\boldsymbol{P}^0} \ \text { (Cholesky factor)}, \boldsymbol{L}_X^{0} = \boldsymbol{0} \in \mathbb {R}^{r \times p}, \nonumber \\ \boldsymbol{U}_{0}= & \boldsymbol{P}_\alpha \equiv \alpha [\boldsymbol{I}^{(*)}][\boldsymbol{I}^{(*)}]^T \end{aligned}$$
  Then, for $$n=1, \cdots , N_T$$:
- *Sampling:* generate 2*p* particles from the current state and parameter estimates, i.e. for i=1,…,2p:
  31b $$\begin{aligned} {\left\{ \begin{array}{ll} \boldsymbol{\hat{X}}^{n-1}_{(i)} = \boldsymbol{\hat{X}}^{n-1}_+ + \boldsymbol{L}_X^{n-1}(\boldsymbol{C}^{n-1})^{T}\boldsymbol{I}_{(i)}, \\ \boldsymbol{\hat{\theta }}^{n-1}_{(i)} = \boldsymbol{\hat{\theta }}^{n-1}_+ + \boldsymbol{L}^{n-1}_{\theta }(\boldsymbol{C}^{n-1})^{T}\boldsymbol{I}_{(i)} \end{array}\right. } \end{aligned}$$
  with $\boldsymbol{C}^{n-1}$ the Cholesky factor of $(\boldsymbol{U}^{n-1})^{-1}$.
- *Prediction:* propagate each particle with the forward model and compute an a priori state prediction:
  31c $$\begin{aligned} {\left\{ \begin{array}{ll} \boldsymbol{\hat{X}}^n_{(i)} = \mathcal {A}^{n}(\boldsymbol{\hat{X}}^{n-1}_{(i)},\boldsymbol{\hat{\theta }}^{n-1}_{(i)}),\quad \boldsymbol{\hat{\theta }}^{n}_{(i)} =\boldsymbol{\hat{\theta }}^{n-1}_{(i)}, \quad i=1,\dots ,2p \\ \boldsymbol{\hat{X}}^n_- = E_\alpha ([(\boldsymbol{\hat{X}}^{n})^{(*)}]) \equiv \alpha \sum _{i=1}^{2p} \boldsymbol{\hat{X}}^n_{(i)} \\ \boldsymbol{\hat{\theta }}^n_- = E_\alpha ([(\boldsymbol{\hat{\theta }}^n)^{(*)}]) \end{array}\right. } \end{aligned}$$
- *Correction:* compute a posteriori estimates based on measurements for state and parameters, using the *i*-th particle innovation $\boldsymbol{\Gamma }^n_{(i)}$:
  31d $$\begin{aligned} {\left\{ \begin{array}{ll} \boldsymbol{L}^n_X = \alpha [(\boldsymbol{\hat{X}}^n)^{(*)}] [\boldsymbol{I}^{(*)}]^{T} \\ \boldsymbol{L}_\theta ^n = \alpha [(\boldsymbol{\hat{\theta }}^n)^{(*)}] [\boldsymbol{I}^{(*)}]^{T} \\ \boldsymbol{L}_\Gamma ^n = \alpha [(\boldsymbol{\Gamma }^n)^{(*)}] [\boldsymbol{I}^{(*)}]^{T} \\ \boldsymbol{U}^n = \boldsymbol{P}_\alpha + (\boldsymbol{L}^n_\Gamma )^{T} \boldsymbol{W}^{-1} \boldsymbol{L}^n_\Gamma \, \quad \boldsymbol{P}_\alpha = \alpha [\boldsymbol{I}^{(*)}] [\boldsymbol{I}^{(*)}]^{T}\\ \boldsymbol{\hat{X}}^n_+ = \boldsymbol{\hat{X}}^n_- - \boldsymbol{L}^n_X (\boldsymbol{U}^n)^{-1}(\boldsymbol{L}^n_\Gamma )^{T} \boldsymbol{W}^{-1} E_\alpha ([(\boldsymbol{\Gamma }^n)^{(*)}]) \\ \boldsymbol{\hat{\theta }}_+^n = \boldsymbol{\hat{\theta }}^n_- - \boldsymbol{L}^n_\theta (\boldsymbol{U}^n)^{-1}(\boldsymbol{L}^n_\Gamma )^{T} \boldsymbol{W}^{-1}E_\alpha ([(\boldsymbol{\Gamma }^n)^{(*)}]) \\ \boldsymbol{P}_{\theta }^{n} = \boldsymbol{L}_{\theta }^{n} (\boldsymbol{U}^n)^{-1} (\boldsymbol{L}_{\theta }^{n})^T\\ \boldsymbol{P}_{X}^{n} = \boldsymbol{L}_{X}^{n} (\boldsymbol{U}^n)^{-1}(\boldsymbol{L}_X^{n})^T \end{array}\right. } \end{aligned}$$

with $$\boldsymbol{W}=\sigma _y^2 \mathbb {I}$$.

## Footnotes

1 From now on, simply referred as *magnetization* for the sake of conciseness.
